# Designing Stress-Adaptive Dense Suspensions Using Dynamic Covalent Chemistry

**DOI:** 10.1021/acs.macromol.2c00603

**Published:** 2022-07-20

**Authors:** Grayson L. Jackson, Joseph M. Dennis, Neil D. Dolinski, Michael van der Naald, Hojin Kim, Christopher Eom, Stuart J. Rowan, Heinrich M. Jaeger

**Affiliations:** † James Franck Institute, University of Chicago, 929 East 57th Street, Chicago, Illinois 60637, United States; ‡ Combat Capabilities and Development Command, Army Research Laboratory, Aberdeen Proving Ground, Maryland 21005, United States; § Pritzker School of Molecular Engineering, University of Chicago, 5640 South Ellis Avenue, Chicago, Illinois 60637, United States; ∥ Department of Physics, University of Chicago, 5720 South Ellis Avenue, Chicago, Illinois 60637, United States; ⊥ Department of Chemistry, University of Chicago, 5735 South Ellis Avenue, Chicago, Illinois 60637, United States; # Chemical and Engineering Sciences Division, Argonne National Laboratory, 9700 Cass Avenue, Lemont, Illinois 60439, United States

## Abstract

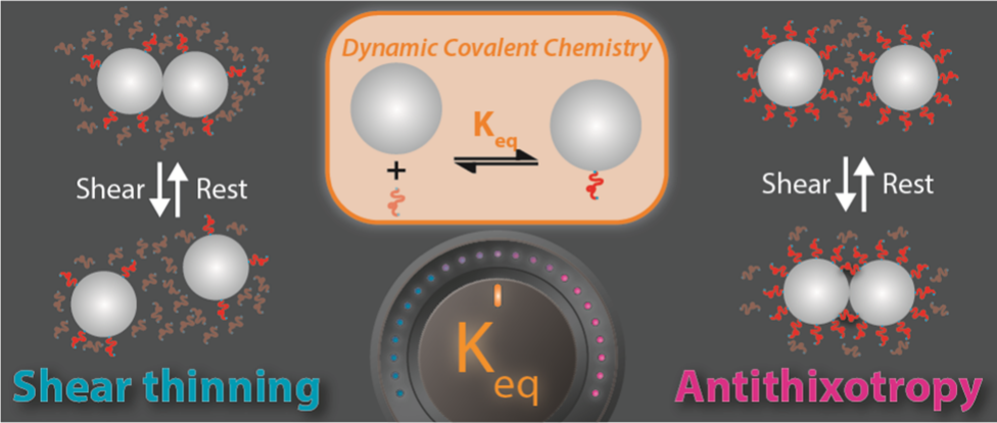

The non-Newtonian behaviors of dense suspensions are central to their use in technological and industrial applications and arise from a network of particle–particle contacts that dynamically adapt to imposed shear. Reported herein are studies aimed at exploring how dynamic covalent chemistry between particles and the polymeric solvent can be used to tailor such stress-adaptive contact networks, leading to their unusual rheological behaviors. Specifically, a room temperature dynamic thia-Michael bond is employed to rationally tune the equilibrium constant (*K*_eq_) of the polymeric solvent to the particle interface. It is demonstrated that low *K*_eq_ leads to shear thinning, while high *K*_eq_ produces antithixotropy, a rare phenomenon where the viscosity increases with shearing time. It is proposed that an increase in *K*_eq_ increases the polymer graft density at the particle surface and that antithixotropy primarily arises from partial debonding of the polymeric graft/solvent from the particle surface and the formation of polymer bridges between particles. Thus, the implementation of dynamic covalent chemistry provides a new molecular handle with which to tailor the macroscopic rheology of suspensions by introducing programmable time dependence. These studies open the door to energy-absorbing materials that not only sense mechanical inputs and adjust their dissipation as a function of time or shear rate but also can switch between these two modalities on demand.

## Introduction

Concentrated or “dense” suspensions of particles in a Newtonian suspending solvent can autonomously sense applied stress and adapt their mechanical properties in response. This feature makes them promising for smart applications in additive manufacturing,^1−3^ coatings and lubrication,^4,5^ vibration dampening,^6^ and impact mitigation.^7,8^ These highly loaded polymer nanocomposites commonly exhibit non-Newtonian flow behaviors, where the viscosity depends on the shear rate or shear stress and may also depend on shearing time. A suspension is termed shear thinning or shear thickening when the viscosity decreases or increases with shear rate, respectively.^9,10^ Additionally, the suspension can be thixotropic if viscosity decreases over time at a fixed shear rate or antithixotropic (also termed negative thixotropic or rheopectic) if it increases over time at a fixed shear rate.^11^ In particular, antithixotropic materials are attractive because they can transform from liquid-like to solid-like under shear, and the resulting properties of the shear-induced gel such as yield stress^12^ and conductivity^13^ can be programmed using their shear history. The ability to controllably tune viscosity (or dissipation) with time would also be relevant to vibration dampening^14^ and impact mitigation. However, antithixotropic materials are rare, and few design rules exist to target this unique and useful non-Newtonian behavior.

The non-Newtonian rheology of dense suspensions originates from microscopic constraints on interparticle motion.^5,10,15−21^ Shear thinning arises from stress-released constraints, which are broken upon increasing shear rate or stress, whereas stress-activated constraints (formed by increasing shear rate or stress) cause shear thickening. While the macroscopic viscosity in either case is constant at a given shear rate or stress, constraints are constantly breaking and re-forming within a structurally dynamic network of noncovalent interparticle contacts. When the viscosity does evolve with time at a constant shear rate as in thixotropy (or antithixotropy), this signals the release (or formation) of constraints.^11^ While great strides have been made in understanding the constraint-based physics of dense suspensions, an emerging challenge is to connect microscopic constraints to specific chemical interactions.^22^

To this end, prior work has focused on manipulating noncovalent chemical interactions such as van der Waals forces, solvation forces,^23−25^ depletion attraction,^26,27^ steric stabilization,^28−31^ and hydrogen bonding.^32−35^ These seminal studies provide a conceptual framework to rationalize basic shear-thickening or shear-thinning behavior in terms of the relative strength of particle–particle and particle–solvent interactions (i.e., solubility).^24,36^ If particle–particle attractions cannot be overcome by particle–solvent interactions, then the suspension possesses adhesive constraints at rest, which are broken by shear (shear thinning). If the particle–solvent interaction strength is increased^24,25,36,37^ and the particle–particle attraction is diminished, e.g., using surfactants^28,29^ or a covalently grafted steric barrier,^30,31^ then particles are dispersed at rest yet can form frictionally stabilized contacts under shear (shear thickening). The understanding from this prior work was established using simple noncovalent interactions, yet synthetic organic chemistry offers nearly limitless potential to tune particle–particle and particle–solvent interactions, thus presenting a new frontier for designing responsive dense suspensions.

Dynamic covalent chemistry (DCC) has recently emerged as a method to engineer stress-adaptive functional polymeric materials.^38−44^ Like the noncovalent interactions described above, dynamic covalent bonds are able to dissociate and re-associate under equilibrium conditions, though they typically require a catalyst or external stimulus to access this reversibility.^42^ When integrated into a dynamic covalent network (DCN) or covalent adaptable network (CAN), dynamic bonds enable structural reorganization under mechanical stress. This behavior is quite sensitive to the dynamic equilibrium constant (*K*_eq_).^39,45^ Past work also used interfacial DCC to integrate functionalized filler particles into cross-linked resins and studied the stress relaxation of these nanocomposites, though the DCCs used required exogenous catalysts.^46−50^ As opposed to these nanocomposite systems with a cross-linked suspending matrix, dense suspensions possess a fluid matrix that allows particle migration and interparticle constraints to be formed or released under shear. While there have been studies of nanoparticle gels stabilized by dynamic covalent cross-links,^51−53^ these works were primarily concerned with self-assembly rather than shear rheology. Within the context of a dense suspension, an ideal DCC would allow ambient temperature dynamic exchange without a catalyst as well as a readily tunable bond strength (*K*_eq_), both of which are achievable using a specific class of thia-Michael (tM) reactions.

The tM reaction is the addition of a thiol to a thia-Michael accepting (tMA) electron-poor olefin to form a thioether adduct.^54^ As demonstrated by foundational small molecule studies,^55−57^ selection of certain substituents adjacent to the double bond can result in catalyst-free dynamic tM bonds at ambient temperatures. Examples of this include benzalcyanoacetate (BCA) and benzalcyanoacetamide (BCAm)-based tMAs. An advantage of BCA or BCAm-based tMAs is that the *K*_eq_ can be tuned by varying the electron-donating/withdrawing nature of the R-substituents attached to the phenyl ring, which has been exploited in dynamic polymer networks,^45^ adhesives,^58^ and hydrogels.^59,60^

**Figure 1:**
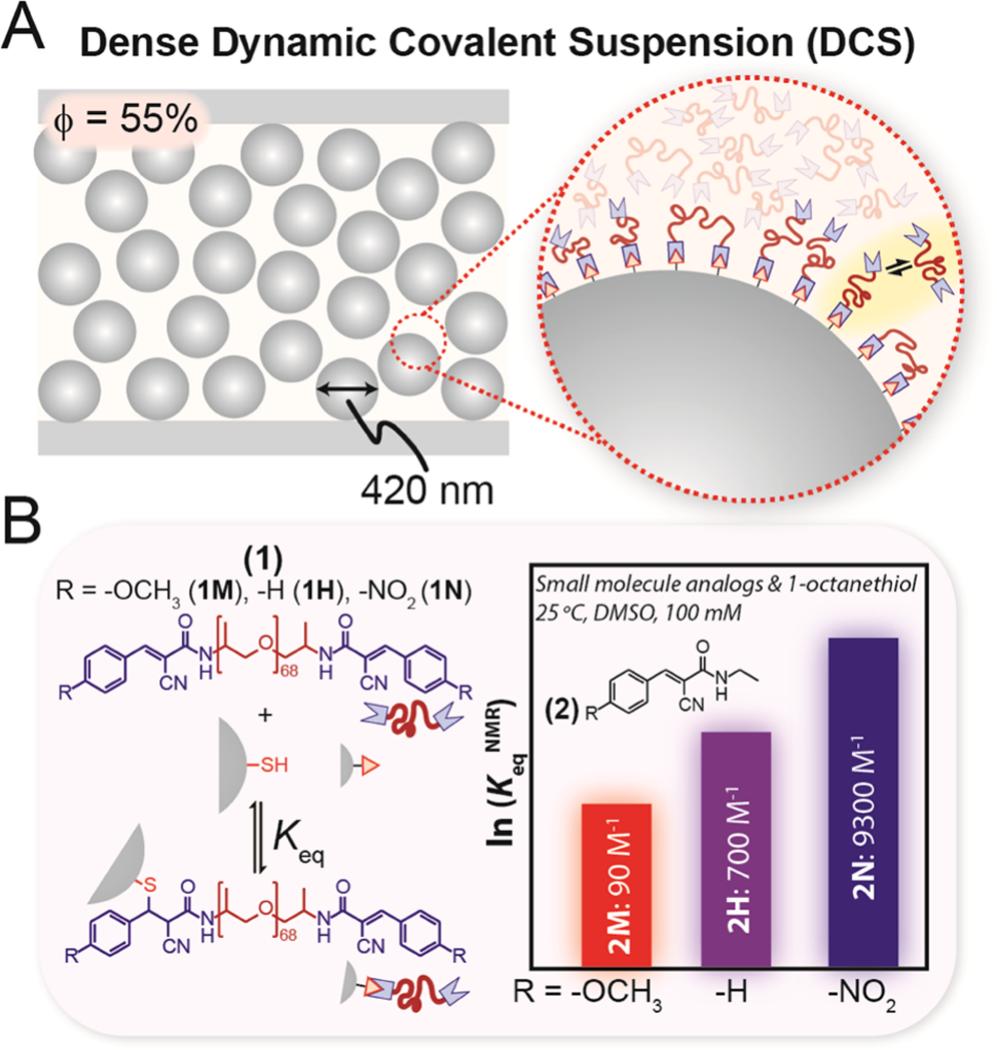
(A) Illustration depicting a dense dynamic covalent suspension (**DCS**). These high volume fraction (ϕ = 55%) **DCSs** contain particles that can form room temperature dynamic covalent bonds with the surrounding fluid polymer matrix, resulting in a bonded polymer graft layer that exchanges dynamically (inset). (B) Realization of **DCSs** using dynamic covalent thia-Michael (tM) chemistry. Chemical structure of ditopic poly(propylene glycol) benzalcyanoacetamide (BCAm) thia-Michael acceptors (tMAs) (**1**) with different R-substituents at the *para*-position of the β-phenyl ring, R = −OCH_3_ (**1M**), −H (**1H**), −NO_2_ (**1N**). These tMAs can form dynamic tM bonds at the surface of thiol-functionalized particles, which exchange dynamically under ambient conditions without a catalyst. Small molecule analogues (**2M**, **2H**, or **2N**) were used to assess how temperature and chemistry affect the dynamic equilibrium constant (*K*_eq_^NMR^).

Taking advantage of the room temperature, catalyst-free, dynamic covalent bonds of the tM reaction with BCAm tMAs, presented here is the first study aimed at exploring the use of DCC in dense suspensions. Specifically, thiol-coated hard-sphere silica particles are dispersed in a fluid matrix composed of a low-molecular-weight BCAm end-capped polymer. Importantly, this polymeric tMA solvent can form dynamic tM bonds at the particle surface to yield a dynamic brush layer (Figure 1A) with a bonding strength (*K*_eq_) orders of magnitude larger than is achievable through a single hydrogen bond. While conventional noncovalent dense suspensions (with only hydrogen bonding interactions at the interface) (**NCSs**) exhibit shear thinning, these dynamic covalent suspensions (**DCSs**) exhibit antithixotropy, wherein the viscosity reversibly increases under shear and relaxes upon shear cessation. Interestingly, the rheology of **DCSs** can be tuned between shear thinning and antithixotropy by varying *K*_eq_ of the tM bond, which in turn affects the dynamic graft density at the particle surface. Moreover, studies of monotopic BCAm tMAs reveal that antithixotropy in **DCSs** arises from partial debonding of the particle grafts from the surface under shear and the formation of polymer bridges between particles.

## Results and Discussion

### Material Synthesis and DCS Preparation

To experimentally realize the concept in Figure 1A, thiol-coated particles were prepared by grafting (3-mercaptopropyl)trimethoxysilane onto commercially available silica particles using literature procedures (Scheme S1).^61^ After surface functionalization, the particle diameter was 417 ± 30 nm (Figure S1), and the particle surface was covered with 0.5 thiols/nm^2^ as determined by NMR.^61^

The tMA end-capped polymer was synthesized in two steps from an amine-terminated *M*_n_ ∼4000 g/mol poly(propylene glycol) (PPG) core. Acid-catalyzed condensation with cyanoacetic acid and subsequent Knoevenagel condensation with different benzaldehydes were used to synthesize three ditopic BCAm polymers (Schemes S2 and S3 and Table S1). These are referred to by their R-substituents at the *para*-position of the β-phenyl ring: methoxy (R = −OCH_3_) (**1M**), unsubstituted (R = −H) (**1H**), and nitro (R = −NO_2_) (**1N**) (Figure 1B).

To understand the baseline effect of temperature and R-substituent on the dynamic equilibrium constant (*K*_eq_), small molecule analogues **2M**, **2H**, and **2N** were synthesized, and NMR was used to measure *K*_eq_^NMR^ under 100 mM equimolar (with 1-octanethiol) conditions in DMSO-*d*_6_ over the temperature range 25–77 °C (Schemes S4 and S5, Figures S2–S7, and Table S2). These experiments show that the value of *K*_eq_^NMR^ can be tuned by over a factor of ∼10^3^ using temperature and chemistry: *K*_eq_^NMR^ decreases upon heating and, at a constant temperature, shows a trend of **2N > 2H** > **2M** (Figure 1B). As the reaction proceeds through a charged enolate intermediate,^57^ reaction rates and overall equilibrium are expected to be impacted by solvent polarity. In addition to this, *K*_eq_ for **DCSs** represents polymeric tMA binding to surface thiols, and the entropic penalty for polymer chain stretching is not accounted for by *K*_eq_^NMR^ and would lead to a lower effective *K*_eq_ in **DCSs**. With respect to these points, the small molecule controls are treated as estimates; however, the trends in temperature and chemistry from the model studies are expected to translate to **DCSs**.

**DCSs** at a silica particle volume fraction (ϕ) of 55% were prepared with either **1M**, **1H**, or **1N** as the suspending solvent to yield **DCS-1M**, **DCS-1H**, or **DCS-1N**. A control noncovalent dense suspension (**NCS-OH**) was prepared at the same ϕ and with the same particles but with 4000 g/mol hydroxy-terminated PPG as the polymer matrix. From ϕ, particle density, and thiol surface coverage it is estimated that [−SH] ∼0.016 M and [tMA] ∼0.46 M in the liquid phase of these suspensions, a nearly 30-fold excess of the tMA. In other words, the surface thiol group is the limiting reagent and leads to a high bonding fraction and subsequent polymer grafting at the particle surface. The tM adducts are envisioned to serve as a dynamic brush layer that depends on the dynamic bond strength (*K*_eq_), with the remaining unbound tMAs serving as the carrier fluid (Figure 1A).

### NCS and DCS Rheology

**Figure 2:**
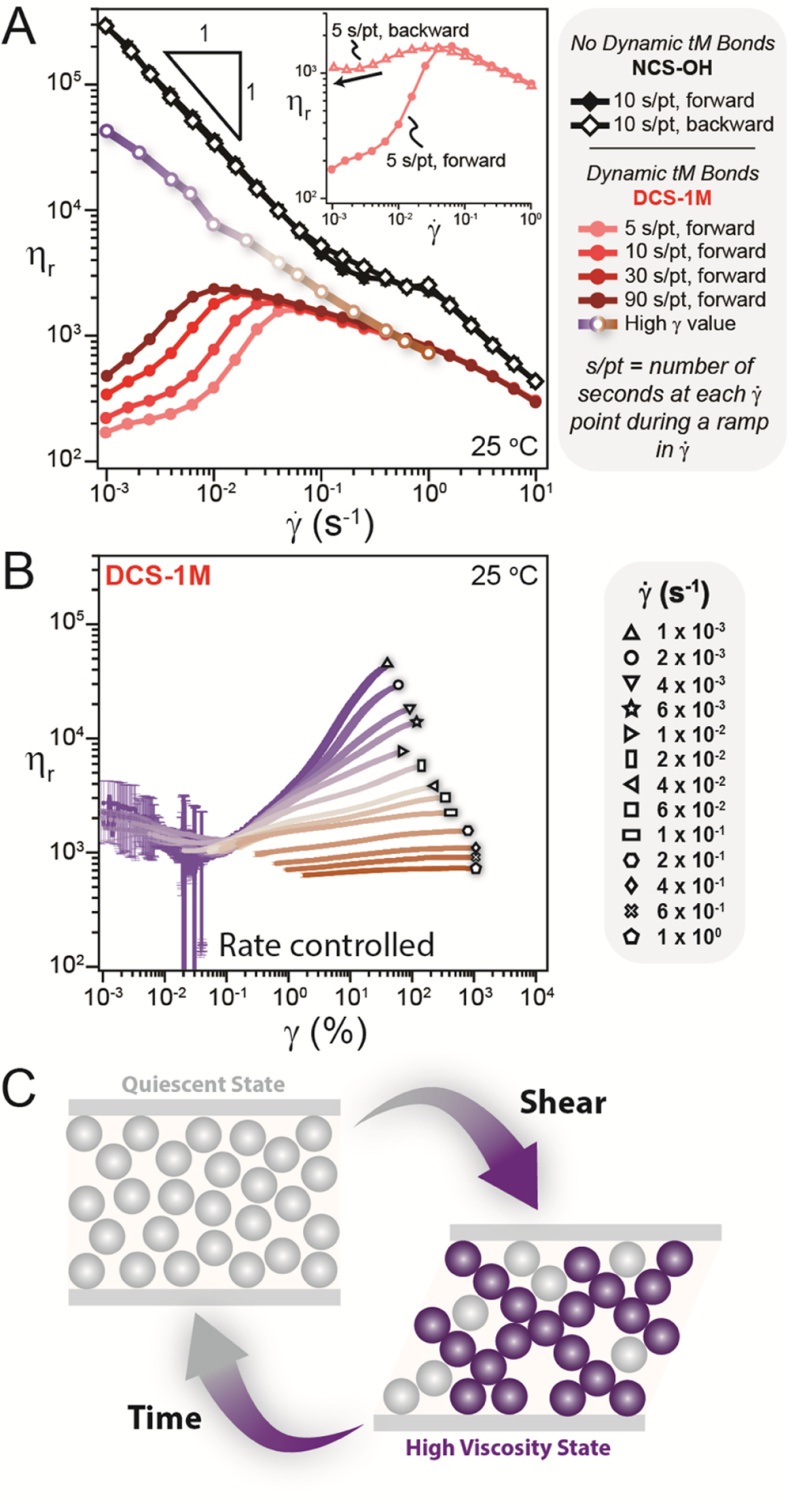
(A) Suspensions with only noncovalent hydrogen bonding interactions between particles and solvent (**NCS-OH**) exhibit reversible shear thinning. In contrast, dynamic covalent suspensions such as **DCS-1M** exhibit antithixotropy. Reduced viscosity η_r_ versus shear rate (γ̇) for **DCS-1M** with different waiting times (s/pt is the number of seconds at each γ̇ point during a ramp in γ̇) reveal that η_r_ increases as a function of shearing time and eventually approaches a steady state. Similarly, a comparison of the increasing (forward) and decreasing (backward) γ̇ ramps reveals hysteresis (inset). The error bars are approximately the size of the plot markers and represent the standard deviation of three forward or backward shear rate sweeps. (B) Evolution of η_r_ as a function of strain (γ) at a constant γ̇. The error bars here represent the standard deviation of the viscosity (see the Supporting Information for details). (C) Schematic depiction of antithixotropy, wherein shear reversibly transforms a low viscosity quiescent state into a higher viscosity state through the formation of a stress-bearing particle network (purple).

**NCS-OH** serves as a useful starting point to understand **DCS** rheology. **NCS-OH** exhibits conventional shear thinning, where the viscosity decreases with increasing shear rate (γ̇) (Figure 2A, black trace). In this figure, the reduced viscosity η_r_ (η_r_ = η_apparent_/η_0_, where η_0_ is the Newtonian viscosity of the suspending solvent and η_apparent_ is the suspension viscosity measured by the rheometer) is used to isolate the viscosity contribution of the particles from that of the suspending polymeric solvent (Figure S8). See the Supporting Information for a discussion of measurement artifacts and Figure S9, which shows all rheology was conducted within measurable limits.^62^ The forward (increasing) and backward (decreasing) shear rate ramps overlay quite well for **NCS-OH**, illustrating no processing hysteresis and viscosity which is independent of shearing time. As explored by others,^19,24,36^ shear-thinning behavior in systems like **NCS-OH** can be understood as a solubility mismatch wherein hydrogen bonding or van der Waals forces between particle and solvent are not strong enough to overcome interparticle attractions. This leads to a stress-bearing (i.e., high viscosity) network of adhesive particle–particle contacts at rest, which is disrupted by shear and leads to shear thinning.^15,29^ These stress-released interparticle contacts rapidly re-form upon shear cessation, and the suspension viscosity does not strongly depend on the shear history.

In stark contrast to the conventional shear thinning of **NCS-OH**, introduction of dynamic tM chemistry at the particle surface in **DCS-1M** leads to rich time-dependent rheology (Figure 2A). The measured viscosity on the forward shear rate ramp is low, while it is much higher upon the backward shear rate ramp, indicating a strong hysteresis (Figure 2A, inset). However, the higher viscosity state decays upon shear cessation. Increasing the waiting time at each point in the shear rate ramp leads to a higher measured viscosity at low shear rates, while the data were identical at higher shear rates (Figure 2A). At a constant shear rate, the viscosity evolves as a function of strain (γ) and shows an initial decay from the presheared state followed by growth and appears to be plateauing to a high γ value (Figure 2B). The viscosity at the largest measured strain, which for γ̇ < 0.1 s^–1^, is an underestimate of the true “steady-state” viscosity, is plotted in Figure 2A and decreases with increasing shear rate. This trend is also apparent when comparing the viscosity for different shear rates at a constant γ value (Figure S10). **DCS-1M** exhibits stronger shear-thinning behavior as γ increases, suggesting a steady-state shear-thinning behavior. This conclusion is also supported by constant stress (creep) measurements of **DCS-1M** revealing a viscosity bifurcation:^12,63^ the viscosity diverges for σ ≤ 10 Pa and flows for σ ≥ 100 Pa (Figure S11). Under shear rate control, this manifests as a viscosity that grows at a constant shear rate and then plateaus to the viscosity at which the applied shear stress is equivalent to the yield stress of the network. In other words, **DCS-1M** exhibits a yield stress but only under shear. Qualitatively similar behavior was observed in constant shear rate and creep measurements of **DCS-1H** (Figure S12) and **DCS-1N** (Figure S13). This reversible increase in viscosity indicates that these **DCSs** are antithixotropic, i.e., the opposite of more conventional thixotropy, where shear forms a stress-bearing particle network that returns to its equilibrium quiescent state upon shear cessation (Figure 2C).^4,11^

Small-amplitude oscillatory shear (SAOS) was used to track the decay of the complex viscosity (η*) to understand how these shear-induced structures in **DCSs** relax upon shear cessation (Figure S14). These SAOS experiments were conducted immediately following the constant shear rate experiments shown in Figures 2B, S12, and S13 (see Figure S14 for experimental protocol). **DCSs** subjected to lower shear rates, which typically had larger η_r_ plateau values prior to SAOS, exhibited a slower decay of η*. In other words, more robust particle contact networks prior to shear cessation typically persisted longer once oscillatory shear was applied. It is worth pointing out that for shear-induced networks with similar η_r_, the decay of η* does not clearly correlate with dynamic bond *K*_eq_ but does coincide with the trend in the viscosity of the dynamic tMA oil matrix (generally the slowest for **DCS-1N**, followed by **DCS-1M**, and then **DCS-1H**). This observation suggests that polymer diffusion also plays a role in the relaxation process.

### Tuning Macroscopic Rheology with *K*_eq_

**Figure 3:**
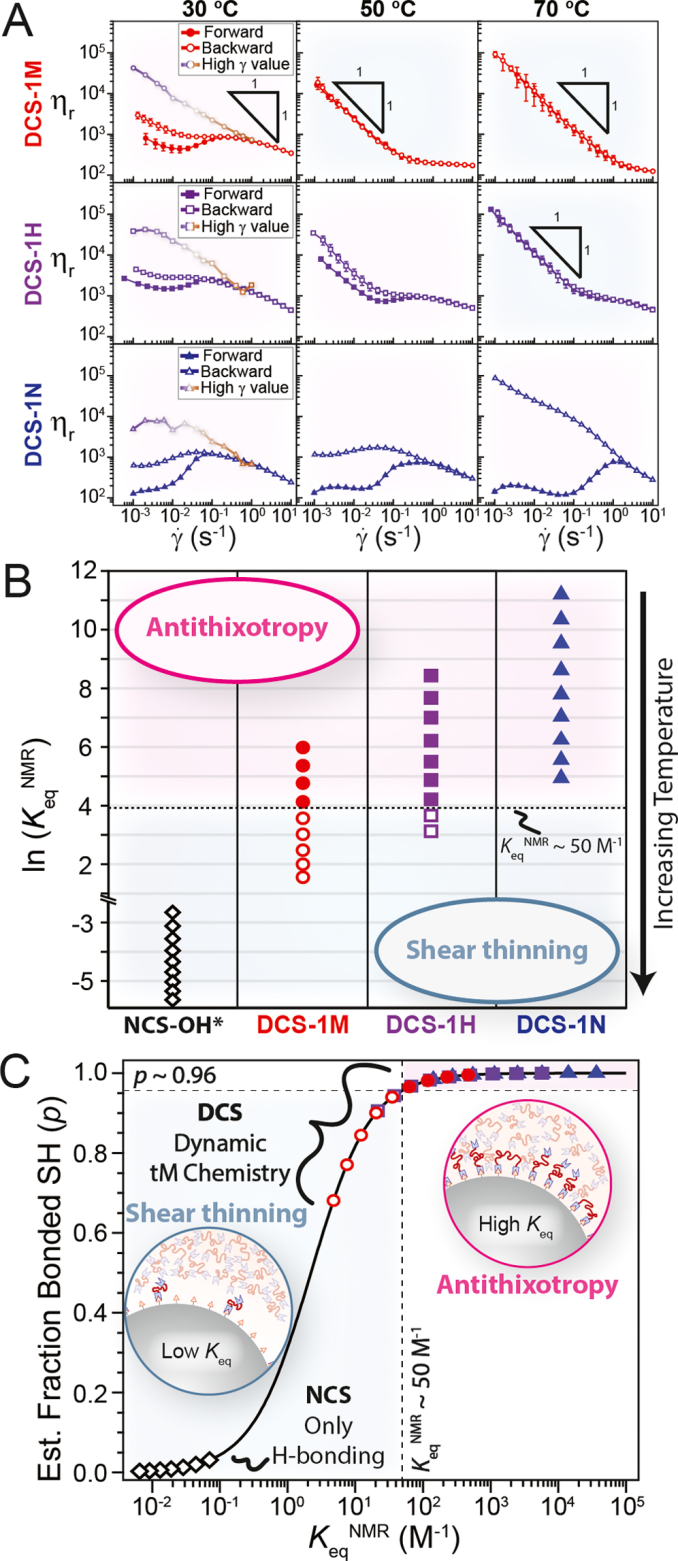
(A) η_r_ versus γ̇ for forward (filled symbols) and backward (open symbols) ramps waiting 10 s at each γ̇ point during a ramp in γ̇ reveal either hysteresis (antithixotropy) or no hysteresis (shear thinning). Error bars for forward–backward shear rate ramps represent the standard deviation of three forward or backward shear rate sweeps. Error bars for the high γ values are approximately the size of the plot markers. (B) Rheological state diagram for 0–80 °C for each system showing a transition from antithixotropy (filled symbols) to shear thinning (open symbols) at *K*_eq_^NMR^ ∼50 M^–1^. *K*_eq_^NMR^ for **NCS-OH** was estimated using literature data (see Table S2). (C) Relating *K*_eq_^NMR^ to an estimated mole fraction of bound thiol (*p*), which serves as a proxy for the brush layer density (solid line, see the Supporting Information for more details). Symbol identities are the same as in (B).

As indicated by the small molecule studies (Figures 1B and S7), *K*_eq_ and the dynamic brush layer density in **DCSs** can be systematically varied using temperature and chemistry. As such, temperature-dependent rheology of **DCSs** was performed over the range 0–80 °C, and shear rate ramps were used to identify antithixotropy or lack thereof (Figures 3A, S15, and S16). These hard-sphere silica particles are considered nonswellable, and thus the particle core volume fraction remains constant over this temperature window. As expected, **NCS-OH** exhibits reversible shear thinning or mild thixotropy over the entire temperature range (Figure S15). However, the **DCSs** are much more sensitive to temperature and changes in *K*_eq_. While **DCS-1M** exhibits antithixotropy at 10 °C (Figure S16) and 30 °C, decreasing *K*_eq_ by heating ≥40 °C leads to strong and reversible shear thinning with a slope of nearly −1 on a log–log plot of η_r_ vs γ̇ (Figure 3A). At 70 °C, it is apparent that η_r_ at a given shear rate is significantly larger even than that reached in the high γ limit at 25 °C. In other words, the stress-bearing particle network formed at rest at 70 °C is stronger than that formed under shear at 25 °C. A similar transition from antithixotropy to shear thinning with mild thixotropy is observed when heating **DCS-1H,** but the transition occurs when heating between 60 and 70 °C (Figure 3A). The area of the hysteresis loop generally decreases upon heating **DCS-1M** or **DCS-1H**, which could reflect a gradual transition between antithixotropy and shear thinning. The larger *K*_eq_ for **DCS-1N** leads to antithixotropy over the entire investigated range with an increasing mismatch between the forward and backward shear rate ramps. This trend for **DCS-1N** is particularly obvious at 70 °C, where the viscosity at the end of the hysteresis loop is 3 orders of magnitude larger than its initial value.

Grouping the data shown in Figure 3A in terms of *K*_eq_^NMR^ determined from small molecule analogues (Figure S7) yields the rheological state diagram shown in Figure 3B. Despite the potential issues in translating *K*_eq_^NMR^ directly to **DCSs** discussed above, the data in Figure 3B show a clear transition between antithixotropy and shear thinning at roughly the same value of *K*_eq_^NMR^ (∼50 M^–1^) whether chemistry or temperature is used as the input variable. **DCS-1N** never reaches a low enough *K*_eq_^NMR^ value to cross the threshold and thus only shows antithixotropy, whereas heating **DCS-1M** or **DCS-1H** leads to shear thinning. Antithixotropy in **DCSs** is accompanied by viscoelastic or liquid-like behavior under SAOS, while shear-thinning **DCSs** are solid-like (Figures S17 and S18). It is worth pointing out that the estimated *K*_eq_^NMR^ value for **NCS-OH** (Table S2) is orders of magnitude below the ∼50 M^–1^ threshold, and this system only exhibits reversible shear thinning.

The effect of *K*_eq_ in **DCSs** and transition from antithixotropy can be understood in terms of changes to the surface grafting density (Figure 3C), which alters particle stability in the surrounding homopolymer matrix in the quiescent, unsheared state. As detailed in the Supporting Information, *K*_eq_^NMR^ can be converted into a fraction of bonded thiols (*p*). Again, this *p*-value likely overestimates the bonding of polymeric tMAs to a surface due to the entropic penalty for polymer chain stretching but serves as a useful proxy for the dynamic graft density in **DCSs** (Figure 3C). Interestingly, *p* shows a precipitous drop in the vicinity of *K*_eq_^NMR^ ∼50 M^–1^, where **DCSs** transition from antithixotropy to shear thinning.

Such a change in nanoparticle dispersibility with grafting density is precedented for covalently grafted polymer brushes. Polymer-grafted nanoparticle (PGNP) stability in a polymer melt depends on grafting density and the relative length of the grafted and matrix polymer chains.^64−66^ In the special case where the graft and matrix polymer chains are the same length (as is the case here), too low of a grafting density leads to partial wetting of the brush by the matrix, and too high of a grafting density causes brush dewetting or a “dry” brush. Both cases lead to particle aggregation due to an entropic depletion attraction. In contrast, intermediate grafting density leads to a wet brush and provides a repulsive barrier and particle dispersal.

Extending these lessons from covalent brushes to dynamic covalent brushes, *K*_eq_ controls the time-averaged graft density (*p*) (Figure 3C). A large *p* leads to an initially dispersed quiescent state, as evidenced by the relatively low initial η_r_ (Figure 3A) and liquid-like or viscoelastic SAOS response (Figures S17 and S18). Decreasing *K*_eq_ below ∼50 M^–1^ leads to a precipitous drop in *p*, which drives particle–particle contacts at rest,^24,29,36^ as evidenced by the higher η_r_, shear thinning under steady shear, and solid-like SAOS response (Figures 3A, S17, and S18).^19,65^ As unbonded surface thiol groups could potentially ionize, we note that the drop in *p* and transition from antithixotropy to shear thinning could also be related to an increase in particle surface charge, which alters nanoparticle dynamics^67^ and stability within the homopolymer melt. The system would be expected to be in the concentrated polymer brush (CPB) regime from a minimum graft density of ∼0.07 chains/nm^2^ to the maximum theoretical graft density of 0.5 chains/nm^2^ set by the interfacial −SH density.^68,69^

The analogy to PGNPs applies to the initial quiescent state of the suspension (i.e., dispersed or aggregated) but does not answer why antithixotropy is observed for **DCSs** under steady shear. Antithixotropy requires shear-induced contacts, which could either be stabilized by polymer bridging or interparticle frictional contacts. Shear-induced polymer bridging would be possible in **DCSs** with ditopic tMAs and has been reported in “shake gels” of small ∼20 nm particles with high molecular weight ∼10^6^ g/mol polymers.^70−74^ On the other hand, antithixotropy is also possible without bridging interactions due to particle–particle frictional contacts, as seen in suspensions with high aspect ratio particles.^12,75,76^ Frictional contacts would be possible in **DCSs** if the dynamic brush were to debond from the particle surface under shear and allow interparticle contacts.

**Figure 4:**
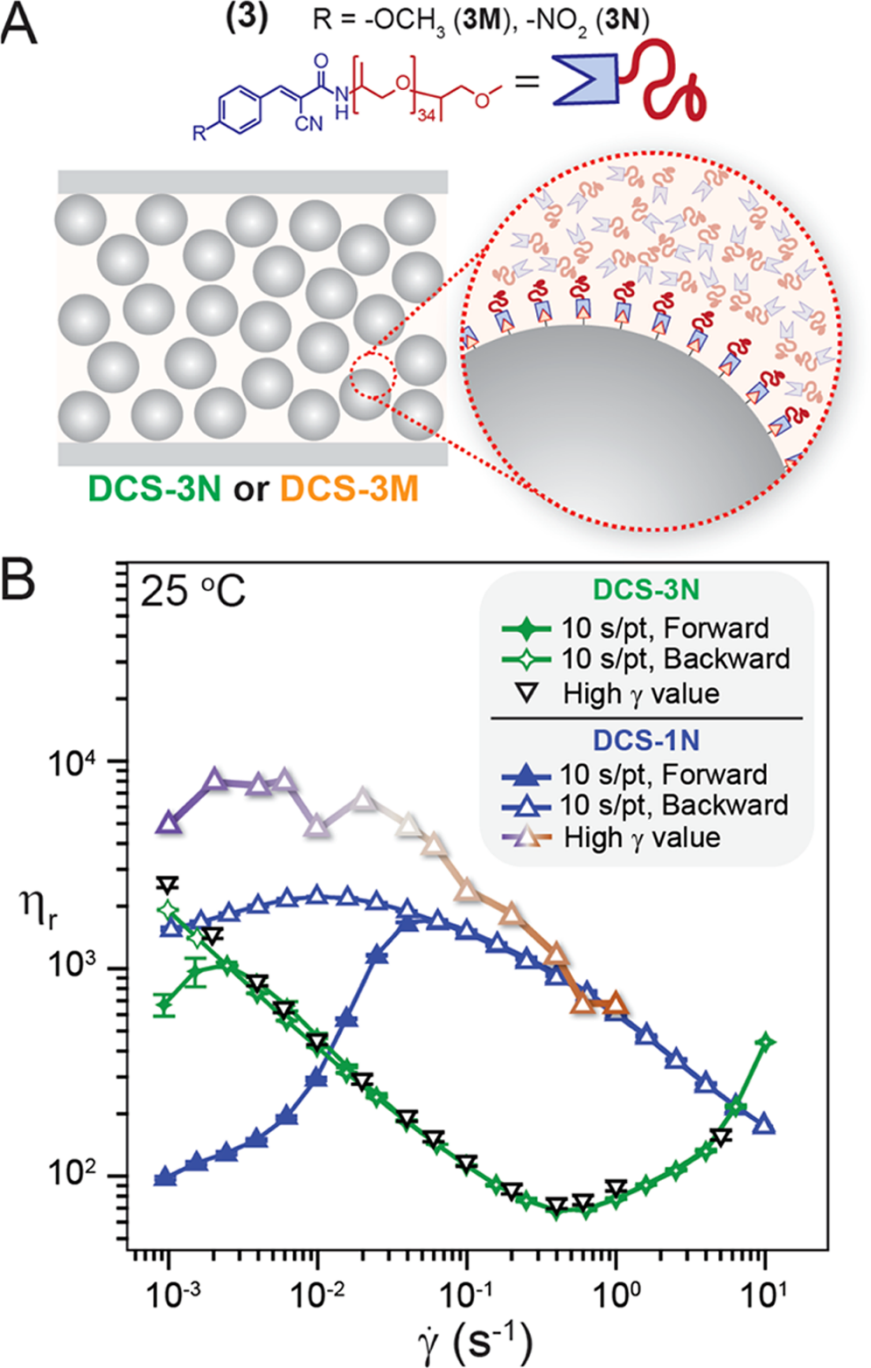
(A) Monotopic tMA **3N** or **3M** leads to **DCSs** without the possibility of shear-induced bridging between particles. (B) Reduced viscosity (η_r_) for a forward–backward shear rate (γ̇) ramp (10 s/pt measurement time) reveals mostly reversible behavior for **DCS-3N**, with a backward shear rate ramp that nearly matches the high γ viscosity (Figure S19). This behavior contrasts greatly with that of the **DCS-1N**, which shows pronounced hysteresis. The high γ reduced viscosity for **DCS-3N** is also much lower than that of **DCS-1N** at a given shear rate. See Figure S19 for a comparison of **DCS-3M** with **DCS-1M**. The error bars (on the order of the marker size) were determined as described in Figures 2 and 3.

To understand whether the primary mechanism for antithixotropy in **DCSs** involves polymer bridging or frictional particle–particle contacts, monotopic tMAs **3M** and **3N** (incapable of bridging) were synthesized (Figure 4A). As the molecular weight of the monotopic **3** is nominally half that of the ditopic **1**, both systems possess roughly the same stoichiometric imbalance of tMA to thiol at a constant solids volume fraction of ϕ = 55%. As shown in Figure 4B, **DCS-3N** exhibits shear thinning at low shear rates, followed by shear thickening past ∼1 s^–1^. Essentially, no hysteresis is observed for **DCS-3N**, and the backward flow curve almost exactly matches the high γ viscosity value (Figure S19). In contrast, the same experimental conditions for **DCS-1N** lead to pronounced hysteresis over a larger shear rate range. The slight hysteresis at low shear rates observed for **DCS-3N** is also observed for a **NCS** prepared with *M*_n_ ∼2000 g/mol hydroxy-terminated PPG (Figure S19), indicating that the dynamic covalent chemistry of monotopic tMAs does not induce hysteresis. Along the same lines, **DCS-3N** and **DCS-3M** equilibrate to their high γ viscosities at orders of magnitude lower strain values (Figure S19) than **DCS-1N** (Figure S13) or **DCS-1M** (Figure 2), respectively. Therefore, the pronounced hysteresis for ditopic tMAs is primarily the result of shear-induced polymer bridging.

Additionally, the ditopic **DCSs** exhibit much larger high γ viscosities than their monotopic counterparts. Even after accounting for the differences in the tMA oil viscosity, the high γ η_r_ for the monotopic **DCSs** at a given shear rate is ∼20 times lower than for the ditopic **DCSs** (Figures 4B and S19). The larger high γ viscosities for ditopic **DCSs** could indicate an additional attractive force between particles,^19^ which would be expected with shear-induced polymer bridging.

**Figure 5:**
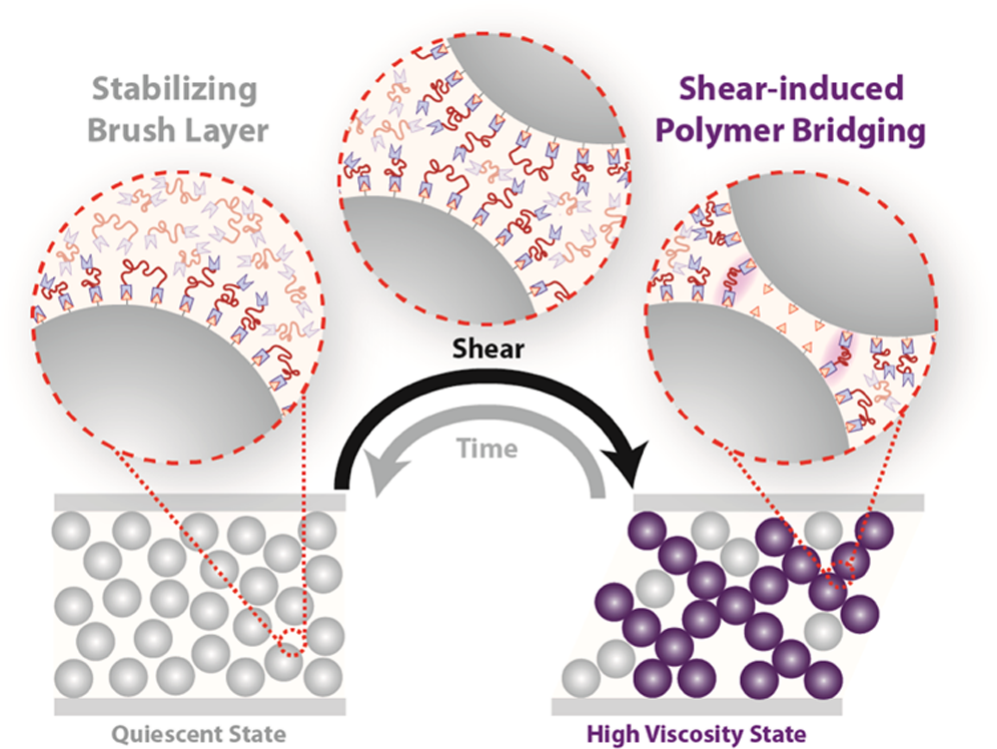
Cartoon illustration of the primary microscopic mechanism for antithixotropy in **DCSs**. Initially dispersed particles are forced into close contact by applied shear, during which the dynamic brush layer can partially debond and enable shear-induced polymer bridges, which stabilize the high viscosity state. Removal of shear regenerates the sterically stabilized low viscosity quiescent state via re-bonding of free tMAs to re-form a repulsive brush layer.

While further experiments are needed to fully understand the effects of dynamic graft molecular weight and stoichiometric imbalance, the comparison between monotopic and ditopic tMAs demonstrates that antithixotropy in ditopic **DCSs** primarily results from shear-induced polymer bridging (Figure 5). Such a mechanism requires not only polymer grafts capable of dynamically bridging between particle surfaces but also exposed (“bare”) surface sites which are dynamically revealed by tM debonding. In this scenario, the increasing hysteresis for **DCS-1N** at elevated temperatures could be explained by a slight reduction in the dynamic graft density (*p*), which allows a larger number of polymer bridges. Prior work on dense suspensions^10^ has shown that contact friction,^77^ surface roughness,^78^ hydrodynamics,^79^ and attraction^26^ contribute to non-Newtonian flow behaviors and could play a role in this system. For instance, Craig and co-workers have demonstrated that tensile force accelerates the dissociation rate in dynamic metal–ligand complexes,^80,81^ meaning that hydrodynamic shear stress at the particle surface could play a role in accelerating tM debonding and exposing surface sites under shear. In other words, *p* may decrease as the shear rate increases. Such tensile forces could also be responsible for the shear-thinning behavior of the antithixotropic networks at high shear rates as a large enough shear stress releases particles from their microscopic tethers before they can re-form. Finally, the decay of the antithixotropic state upon shear cessation (Figure S14) correlates with the viscosity of the particle network before decay, with a lesser dependence on the viscosity of the dynamic tMA oil matrix. These data suggest that once the polymer bridges have stabilized the shear-induced network, contact relaxation requires particles to separate and subsequent reinfiltration of free tMA polymers to regenerate the repulsive brush layer (Figure 5).

## Conclusions

The use of dynamic covalent thia-Michael chemistry at the particle–solvent interface has been shown to be a new approach to control the macroscopic flow behavior of dense suspensions. Small molecule control experiments were used to understand how temperature and chemistry control the equilibrium bonding constant (*K*_eq_). **DCSs** with a high *K*_eq_ exhibit antithixotropy, a rare non-Newtonian behavior, where viscosity increases with shearing time and relaxes upon shear cessation. Decreasing *K*_eq_ in these **DCSs** led to more conventional rheology such as shear thinning. The changes in rheology with *K*_eq_ are interpreted in terms of the polymer graft density at the particle surface and subsequent wetting behavior by the surrounding homopolymer matrix. Finally, monotopic tMAs were used to elucidate the primary mechanism of **DCS** antithixotropy, namely, that the dynamic covalent brush layer partially debonds under shear to enable polymer bridges between particles.

Incorporation of dynamic covalent grafts at a particle surface provides a new path forward toward the general design of antithixotropic materials, which can controllably adjust their dissipation over time in response to mechanical inputs. Furthermore, the tunability of this particular system enables temperature to control the polymer graft density *in situ* and further toggle between two types of non-Newtonian behaviors, antithixotropy and shear thinning. This fine level of control over energy absorption and dissipation opens the door to new materials for vibration dampening, shock absorption, and impact mitigation.

## Supporting Information Available

Experimental details, synthetic schemes, suspension preparation, small molecule control experiments, creep and constant shear rate measurements, decay of the antithixotropic state, temperature-dependent rheology for **NCS-OH**, and SAOS data and SAOS state diagram for all ditopic systems (PDF)

## Notes

Safety statement: no unexpected or unusual safety hazards were encountered in the work reported.
