# Supplementary material for: Designing Stress-Adaptive Dense Suspensions Using Dynamic Covalent Chemistry
Source: Macromolecules. 2022 Jul 20;55(15):6453–61. doi: 10.1021/acs.macromol.2c00603 (PMC9367004; doi:10.1021/acs.macromol.2c00603)
Supplement: Supplementary file 1 — ma2c00603_si_001.pdf [file ma2c00603_si_001.pdf]

Supporting Information

Designing Stress-Adaptive Dense Suspensions using Dynamic Covalent Chemistry

Grayson L. Jackson,[a,*] Joseph M. Dennis,[b] Neil D. Dolinski,[c] Michael van der Naald,[a,d]

Hojin Kim,[a,c] Christopher Eom,[c] Stuart J. Rowan,[c,e,f] and Heinrich M. Jaeger[a,d]

[a] *James Franck Institute, University of Chicago, 929 E. 57th Street, Chicago, Illinois 60637, United States*

[b] *Combat Capabilities and Development Command, Army Research Laboratory, Aberdeen Proving Ground, MD 21005, USA*

[c] *Pritzker School of Molecular Engineering, University of Chicago, 5640 S. Ellis Avenue, Chicago, Illinois 60637, United States*

[d] *Department of Physics, University of Chicago, 5720 S. Ellis Avenue, Chicago, Illinois 60637, United States*

[e] *Department of Chemistry, University of Chicago, 5735 S Ellis Avenue, Chicago, Illinois 60637, United States*

[f] *Chemical and Engineering Sciences Division, Argonne National Laboratory, 9700 Cass Avenue, Lemont, Illinois 60439, United States*

*Corresponding author: graysonjacksonphd@gmail.com

Table of Contents	Page
**I. Suspension Materials, Characterization and Preparation**	3-16
Preparation of thiol-coated silica particles (Scheme S1)	4
**Figure S1.** SEM of thiol-coated particles	5
Polymeric thia-Michael Acceptor (tMA) (**1** & **3**) Synthesis (Schemes S2-3) and Characterization (Table S1)	6-7
Small Molecule tMA (**2**) Synthesis (Schemes S4-5)	8-9
**Figure S2**. NMR spectra for 2-cyano-N-ethylacetamide	10
**Figure S3.** NMR spectra for **2M**	11
**Figure S4.** NMR spectra for **2H**	12
**Figure S5.** NMR spectra for **2N**	13
Determination of K_{eq}^{NMR} for small molecule tMAs (**2M**, **2H**, and **2N**) and 1-octanethiol	14
**Figure S6.** NMR spectra for association of **2M** and 1-octanethiol	15
**Figure S7.** K_{eq}^{NMR} as a function of temperature and tMA chemistry	16
**Table S2.** Results from van't Hoff analysis of small molecules (**2M**, **2H**, and **2N**)	16
**II. Suspension Preparation and Estimation of fraction bonded -SH (*p*)**	17
**III. Rheological Characterization**	18-26
**Figure S8.** Temperature dependent viscosity (η_0) of polymeric solvents	18
**Figure S9.** Identifying the measurable limits of shear rheometry	20
**Figure S10.** Evolution of **DCS** viscosity at a constant strain	21
**Figure S11.** Constant Shear Stress (*creep*) measurements of **DCS-1M**	22
**Figure S12.** Constant Shear Rate and Creep measurements of **DCS-1H**	22
**Figure S13.** Constant Shear Rate and Creep measurements of **DCS-1N**	23
**Figure S14.** Decay of High Viscosity Antithixotropic State	24
**Figure S15.** Temperature dependent hysteresis loops for non-dynamic **NCS-OH** showing only reversible shear thinning or mild thixotropy	25
**Figure S16.** Hysteresis loops for **DCSs** at 10 °C	26
**Figure S17.** Temperature dependent small amplitude oscillatory shear (SAOS) for **DCSs** and **NCS-OH**	27
**Figure S18.** SAOS rheological state diagram as a function of K_{eq}^{NMR}	28
**Figure S19.** Comparison between **DCS-3M** and **DCS-1M** and constant shear rate measurements for **DCS-3M** and **DCS-3N**	29
**IV. References**	30

I. Suspension Materials, Characterization, and Preparation

Materials. 500 nm silica particles (AngstromSphere Silica Microspheres) were purchased from Fiber Optic Center Inc. (New Bedford, MA, USA) and used as received. All other reagent grade solvents were purchased from Sigma-Aldrich (Milwaukee, WI, USA) and used as received.

Characterization. *Scanning Electron Microscopy (SEM):* SEM images were acquired using a Carl Zeiss-Merlin field emission scanning electron miscroscope housed in the Materials Preparation and Measurement Laboratory of the University of Chicago MRSEC operating at an accelerating voltage of 5.0 kV and using the Everhart-Thornley secondary electron detector.

Nuclear Magnetic Resonance (NMR): NMR data for dissolved particles were acquired on a 400 MHz Bruker AVANCE III HD nanobay spectrometer equipped with a BBFO SmartProbe and 24-sample SampleCase autosampler, using Topspin 3.6.2. NMR of polymer samples and variable temperature data were performed using a 500 MHz Bruker AVANCE III HD 500; 11.7 Tesla NMR at the NMR facilities at the University of Chicago. Spectra were referenced using residual protiated solvent peaks. For experimental temperatures ranging 25–40 °C, a standard plastic sample spinner was used; for equilibrium measurements at 50 °C and up, the sample was held in a ceramic spinner (to avoid spinner warpage). Sample temperatures were measured by proxy through ethylene glycol standards via procedures published previously.[1, 2]

Size Exclusion Chromatography (SEC): A Shimadzu HPLC operating in conjunction with a Wyatt Dawn HELEOS II multi-angle light scattering calibrated with polystyrene standards was used to determine the relative molecular weights and distributions. The eluent was THF, and the columns were an inline pair of Agilent PLgel 5 um MIXED-D columns heated to 30 °C.

Differential Scanning Calorimetry (DSC): DSC was performed using a TA Instruments Discovery 2500 Differential Scanning Calorimeter equipped with a refrigeration cooling unit at the Soft Matter Characterization Facility at the University of Chicago. Samples were prepared in T-zero aluminum pans from TA Instruments. A heat-cool-heat cycle was used to eliminate thermal history of the sample and the second heat was used to determine the glass transition temperature as the midpoint of the step transition. Temperature ramps for the heat cycles were 10 °C/min, while the cooling rate was -80 °C/min.

IA. Synthetic Methods

Preparation of Thiol-Coated Silica Particles

Mercaptopropyl groups were grafted onto the particle surfaces using a procedure adapted from Crucho *et al.*[3] In a 2 L flask fitted with a reflux condenser, Silica particles (15.9 g) were dispersed via sonication in 800 mL of toluene that had been dried over $MgSO_4$(*s*). To this 240 mL of (3-mercaptopropyl)trimethoxysilane (10-fold stoichiometric excess) was added and heated to reflux for 24 hr under a N_2 atmosphere. The thiol-functionalized silica NPs were isolated via centrifugation and then redispersed in EtOH. After redispersing in EtOH and centrifuging 5 times, the supernatant tested negative for free thiols using 5,5'-dithiobis(2-nitrobenzoic acid) (*Ellman's reagent*). Particle density was determined to be 1.92 +/- 0.01 g/mL by dispersing the particles in a series of bromoform:methanol mixtures and centrifuging to see whether the particles floated or sank.

Scheme S1. Synthesis of thiol-coated silica particles

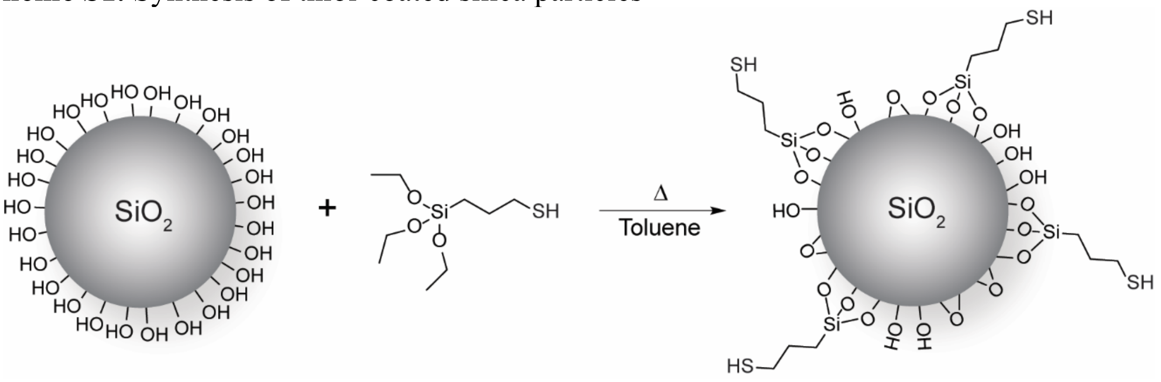

The thiol surface functional group density was determined by dissolving 25 mg of particles in 0.75 mL of 0.5 M NaOD in D_2O at 90 °C and using 1,3,5-trixoane as an internal standard to measure the intensity of the $HSC\underline{H}_2CH_2CH_2Si(ONa)_3$ peak at 2.82 ppm,[3] which yielded a value of 6.7 μmol SH/g particle. Using the measured particle density and assuming an idealized spherical particle, the surface density of thiol groups was calculated as 0.5 groups/nm^2 (~0.9 μmol/m^2) which is the same as that reported by Crucho *et al.*[3] and comparable to other reported values.[4] Considering the tridentate binding of the mercaptopropyl groups and a hydroxl surface group density of 5/nm^2,[5] our calculated value indicates that the thiol surface coverage is ~40% of its theoretical maximum as depicted schematically in Scheme S1. Note that the much smaller surface area to volume ratio for these relatively large, 417 nm diameter particles meant that surface group quantitation by FTIR[4] did not yield a discernable signal and mass loss from TGA of thiol-coated particles was indistinguishable from that due to silica network condensation in unfunctionalized particles.[3]

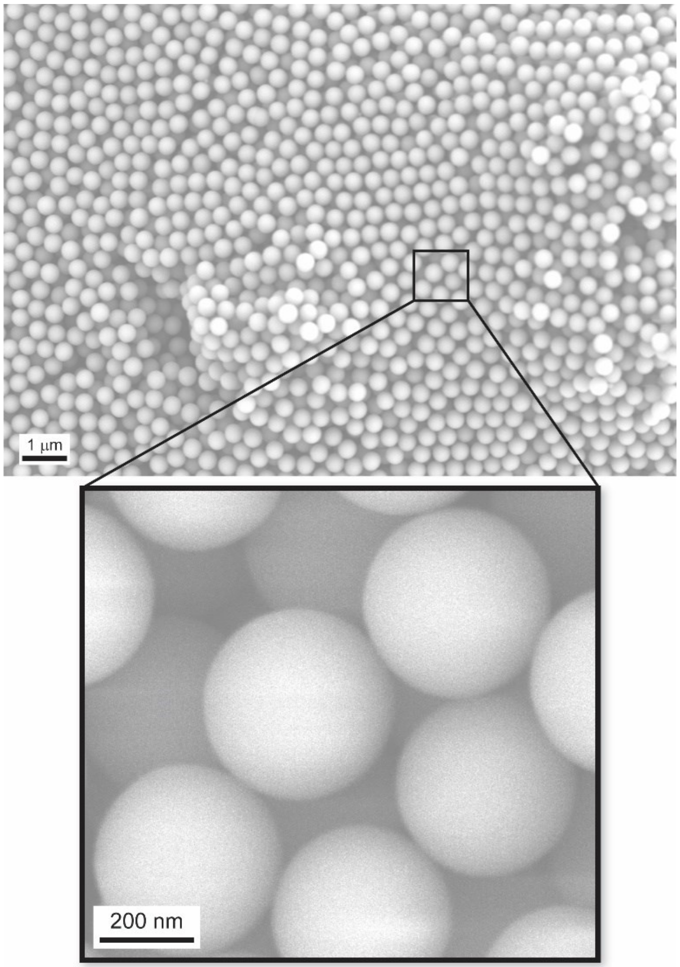

Figure S1. SEM of thiol-coated particles reveal nominally smooth particles with $d = 417 \pm 30$ nm.

Synthesis of Ditopic (1M, 1H, 1N) or Monotopic (3M, 3N) Polymer tMAs for Rheology

Scheme S2. Synthesis of Cyanoacetamide-terminated Polypropylene Glycol

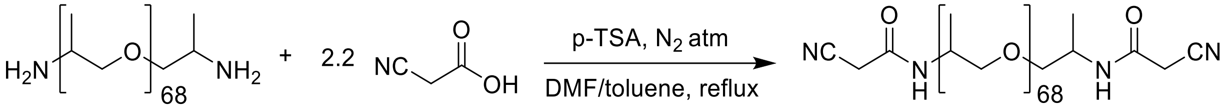

Amine-terminated poly(propylene glycol), (Jeffamine D-4000), was used as received from Sigma-Aldrich (starting material for **1M**, **1H**, **1N**) Monofunctional Jeffamine M-2005 was supplied by the Huntsman Corporation (starting material for **3M**, **3N**). The synthetic protocols were similar for both starting materials so the functionalization of Jeffamine D-4000 is used as a representative example. Jeffamine D-4000 (10 g, 2.5 mmol), *para*-toluene sulfonic acid (*p*-TSA) (0.1 g) and cyanoacetic acid (0.47 g, 5.5 mmol) was added to a 100-mL, round-bottomed, two-necked flask equipped with a magnetic stir bar, nitrogen inlet, temperature probe and Dean-stark trap with condenser. Dimethylformamide (DMF) (10 mL) and toluene (30 mL) were added to the flask and the contents were purged with nitrogen for 10 minutes at room temperature. Following the nitrogen purge, the homogeneous solution was then heated to a reflux temperature of 110 °C, as measured by the temperature probe. The reaction proceeded under reflux until the azeotropic removal of water into the Dean-stark trap ceased (approximately 4 hours). In general, the quantitative measure of water removal corresponded well to the expected reaction conversion with an additional amount of water likely resulting from water contamination of the starting materials (e.g. DMF and PPG). After 4 hours, toluene was then distilled from the reaction flask until the solution temperature reached 120 °C (approximately 30 min). The resulting homogenous, pale yellow solution was then cooled to room temperature and diluted with 50 mL of chloroform. Three washes of a saturated $NaHCO_3$(*aq*) solution, followed by three washes with deionized water (100 mL each wash) were used to remove the residual *p*-TSA and DMF. Finally, the organic phase was then dried with $MgSO_4$(*s*), filtered, and the remaining chloroform was removed using a rotary evaporator.

Yield: 9.2 g (92%) of a pale yellow/orange viscous oil

Scheme S3. Synthesis of Ditopic (**1M**, **1H**, **1N**) or Monotopic (**3M, 3N**) tMA (Benzalcyanoacetamide-terminated Polypropylene Glycol)

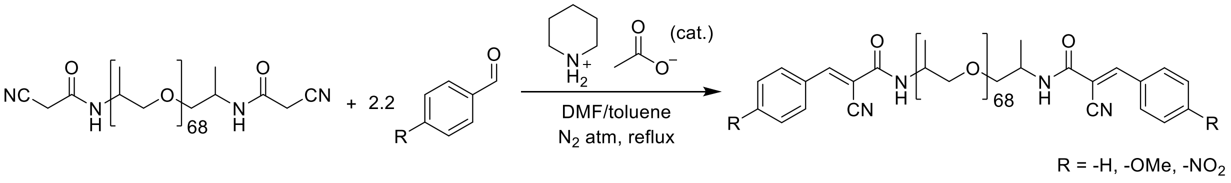

Conversion of the cyanoacetamide-terminated PPG to a ditopic (**1M**, **1H**, **1N**) or monotopic (**3M, 3N**) benzalcyanoacetamide-terminated poly(propylene glycol) followed a similar procedure described by Cope.[6] As a representative example, the synthesis of the unsubstituted benzalcyanoacetamide-terminated PPG is described. Cyanoacetamide-terminated poly(propylene

glycol) (10 g, 2.5 mmol), benzaldehyde (0.58 g, 5.5 mmol) and piperdinium acetate (0.1 g) were added to a 100-mL, round-bottomed, two-necked flask equipped with a magnetic stir bar, nitrogen inlet, temperature probe, and Dean-stark trap with condenser. DMF (10 mL) and toluene (30 mL) were added to the flask and the contents were purged with nitrogen for 10 minutes at room temperature. Following the nitrogen purge, the homogeneous solution was then heated to a reflux temperature of 110 °C, as measured by the temperature probe. The reaction proceeded under reflux until the azeotropic removal of water into the Dean-stark trap ceased (approximately 4 hours). After 4 hours, toluene was then distilled from the reaction flask until the solution temperature reached 120 °C (approximately 30 min). The resulting homogenous, pale orange solution was then cooled to room temperature and diluted with 50 mL of chloroform. Three washes of a saturated sodium bicarbonate solution, followed by three washes with deionized water (100 mL each wash) removed the residual benzaldehyde and DMF. Finally, the organic phase was then dried with $MgSO_4$(*s*), filtered, and the remaining chloroform was removed using a rotary evaporator. Products were recovered as a pale orange/brown viscous oils.

1M: Recovered Yield: 82% **^{1}H NMR** (500 MHz, $CDCl_3$): δ = 8.26 (s, 1H, C=CH), 7.72 (d, 2H, Ar-H), 6.93 (d, 2H, Ar-H), 3.87 (s, 3H, Ar-OCH_3), 3.36-3.66 (m, -CH_2-CH-), 1.26 (m, -CH_3) ppm.

1H: Recovered Yield: 86% **^{1}H NMR** (500 MHz, $CDCl_3$): δ = 8.34 (s, 1H, C=CH), 7.75 (t, 3H, Ar-H), 7.48 (d, 2H, Ar-H), 3.36-3.66 (m, -CH_2-CH-), 1.26 (m, -CH_3) ppm.

1N: Recovered Yield: 75% **^{1}H NMR** (500 MHz, $CDCl_3$): δ = 8.46 (s, 1H, C=CH), 8.26 (d, 2H, Ar-H), 7.91 (d, 2H, Ar-H), 3.36-3.66 (m, -CH_2-CH-), 1.26 (m, -CH_3) ppm.

3M: Recovered Yield: 85% **^{1}H NMR** (500 MHz, $CDCl_3$): δ = 8.26 (s, 1H, C=CH), 7.68 (d, 2H, Ar-H), 6.91 (d, 2H, Ar-H), 3.86 (s, 3H, Ar-OCH_3), 3.36-3.66 (m, -CH_2-CH-), 1.26 (m, -CH_3) ppm.

3N: Recovered Yield: 71% **^{1}H NMR** (500 MHz, $CDCl_3$): δ = 8.39 (s, 1H, C=CH), 8.26 (d, 2H, Ar-H), 7.90 (d, 2H, Ar-H), 3.36-3.66 (m, -CH_2-CH-), 1.15 (m, -CH_3) ppm.

Table S1. Polymer Characterization

	**GPC**		**NMR**	**T_g**
Sample	M_n (x 10^3 g/mol)	Đ	MW (x 10^3 g/mol)	°C
**1M**	6.3	1.51	6.5	-67
**1H**	6.2	1.52	6.3	-67
**1N**	5.7	1.48	7.0	-65
**3M**	3.6	1.16	3.4	-70
**3N**	4.0	1.10	2.7	-72

Synthesis of Small Molecule tMAs (2) for NMR Experiments:

Scheme S4. Synthesis of 2-cyano-N-ethylacetamide precursor:

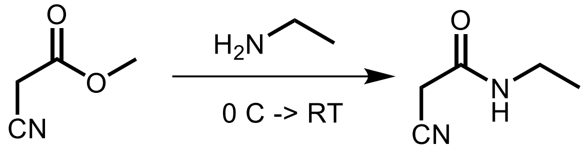

Synthesis adapted from Ref[7]. Methyl cyanoacetate (9.9 g, 99.9 mmol) was added to a 250 mL flask and 15 mL of ethyl amine solution (68-72% in water) was added dropwise (~15 minutes to completely add) at 0 °C. After addition, the ice bath was removed and the reaction mixture was allowed to stir for 12 hours. The mixture was then evaporated under reduced pressure to yield a thick, dark brown oil. At this point, 20 mL of ether and 10 mL of hexanes were added, precipitating an orange solid. The mixture was then filtered and washed with 2:1 ether:hexanes to yield the desired solid product, which was used without further purification.

Yield: 10 g (89%) of an orange solid
^{1}H NMR (500 MHz, $CDCl_3$): δ 7.20 (s, 1H, -NH-), 3.44 (s, 2H, -CH_2-), 3.30 – 3.20 (m, 2H, -CH_2-), 1.12 (t, J = 7.3 Hz, 3H, -CH_3) ppm.
^{13}C NMR (126 MHz, $CDCl_3$): δ 161.83, 115.20, 35.22, 25.95, 14.28 ppm.

Scheme S5. Synthesis of 2R from 2-cyano-N-ethylacetamide

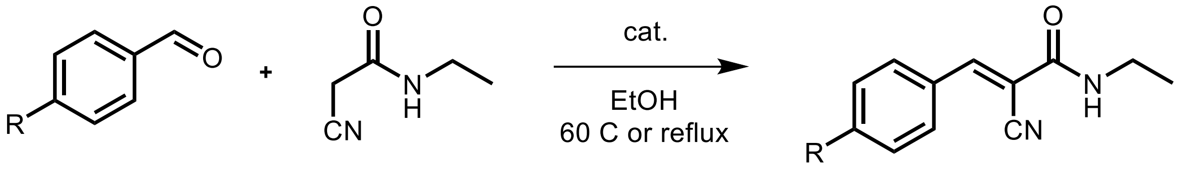

Synthesis of 2M:

2-cyano-N-ethylacetamide (0.5 mg, 4.46 mmol) and *p*-anisaldehyde (542 µL, 4.46 mmol) were dissolved in 20 mL of ethanol. The reaction was heated to reflux, at which point ~20 mg of piperidinium acetate was added to the solution and was left to react for 12 hours. The reaction was then cooled and the solvent was evaporated. The residue was redissolved in methanol and placed in a freezer (-20 °C) for several hours. Water was added to the cooled solution (~10-20% volume) until a precipitate formed. The mixture was then filtered and washed with 1:1 water:methanol to yield the desired solid product.

Yield: 350 mg (33%) of an off-white solid
^{1}H NMR (500 MHz, $CDCl_3$): δ 8.25 (s, 1H, C=CH), 7.93 (d, J = 8.9 Hz, 2H, Ar-H), 6.98 (d, J = 8.9 Hz, 2H, Ar-H), 6.33 (s, 1H, -NH-), 3.88 (s, 3H, -CH_3), 3.47 (qd, J = 7.3, 5.6 Hz, 2H, -CH_2-), 1.24 (t, J = 7.3 Hz, 3H, -CH_3) ppm.
^{13}C NMR (126 MHz, $CDCl_3$): δ 163.35, 160.75, 152.27, 133.12, 124.80, 117.88, 114.77, 100.68, 55.66, 35.54, 14.80, 14.79 ppm.

Synthesis of 2H:

2-cyano-*N*-ethylacetamide (1.0515 g, 9.3775 mmol) and benzaldehyde (953.2 µL, 9.3775 mmol) were combined with piperidine (926.33 µL, 9.3775 mmol) in 282.66 mL ethanol. The reaction mixture was stirred for 12 h at 60 °C, and the solvent was evaporated. The residue was purified by column chromatography on silica gel (CH_2Cl_2).

Yield: 710.6 mg (38%) of a bright yellow solid
^{1}H NMR (500 MHz, $CDCl_3$): δ 8.34 (s, 1H, C=CH), 7.93 (d, *J* = 6.8 Hz, 2H, Ar-H), 7.57 – 7.45 (m, 3H, Ar-H), 6.41 (s, 1H, -NH-), 3.53 – 3.44 (m, 2H, $-CH_2-$), 1.26 (t, *J* = 7.3 Hz, 3H, $-CH_3$).
^{13}C NMR (126 MHz, $CDCl_3$) δ 160.10, 152.94, 132.79, 131.97, 130.68, 129.32, 117.20, 104.21, 35.64, 14.74.

Synthesis of 2N:

2-cyano-N-ethylacetamide (0.5 mg, 4.46 mmol) and 4-nitrobenzaldehyde (674 mg, 4.46 mmol) were dissolved in 20 mL of ethanol. The reaction was heated to reflux, at which point ~20 mg of piperidinium acetate was added to the solution and was left to react for 12 hours. The reaction was then cooled, and the solvent was evaporated. The residue was redissolved in methanol and placed in a freezer (-20 °C) for several hours. Water was added to the cooled solution (~10-20% volume) until a precipitate formed. The mixture was then filtered and washed with 1:1 water:methanol to yield the desired solid product.

Yield: 450 mg (41%) of a pale-yellow solid
^{1}H NMR (500 MHz, $CDCl_3$): δ 8.39 (s, 1H, C=CH), 8.33 (d, *J* = 8.8 Hz, 2H, Ar-H), 8.06 (d, *J* = 8.8 Hz, 2H, Ar-H), 6.47 (d, *J* = 5.8 Hz, 1H, -NH-), 3.50 (qd, *J* = 7.3, 5.6 Hz, 2H, $-CH_2-$), 1.27 (t, *J* = 7.3 Hz, 3H, $-CH_3$) ppm.
^{13}C NMR (126 MHz, $CDCl_3$): δ 158.94, 149.81, 149.52, 137.57, 131.17, 124.41, 116.18, 108.53, 35.87, 14.65 ppm.

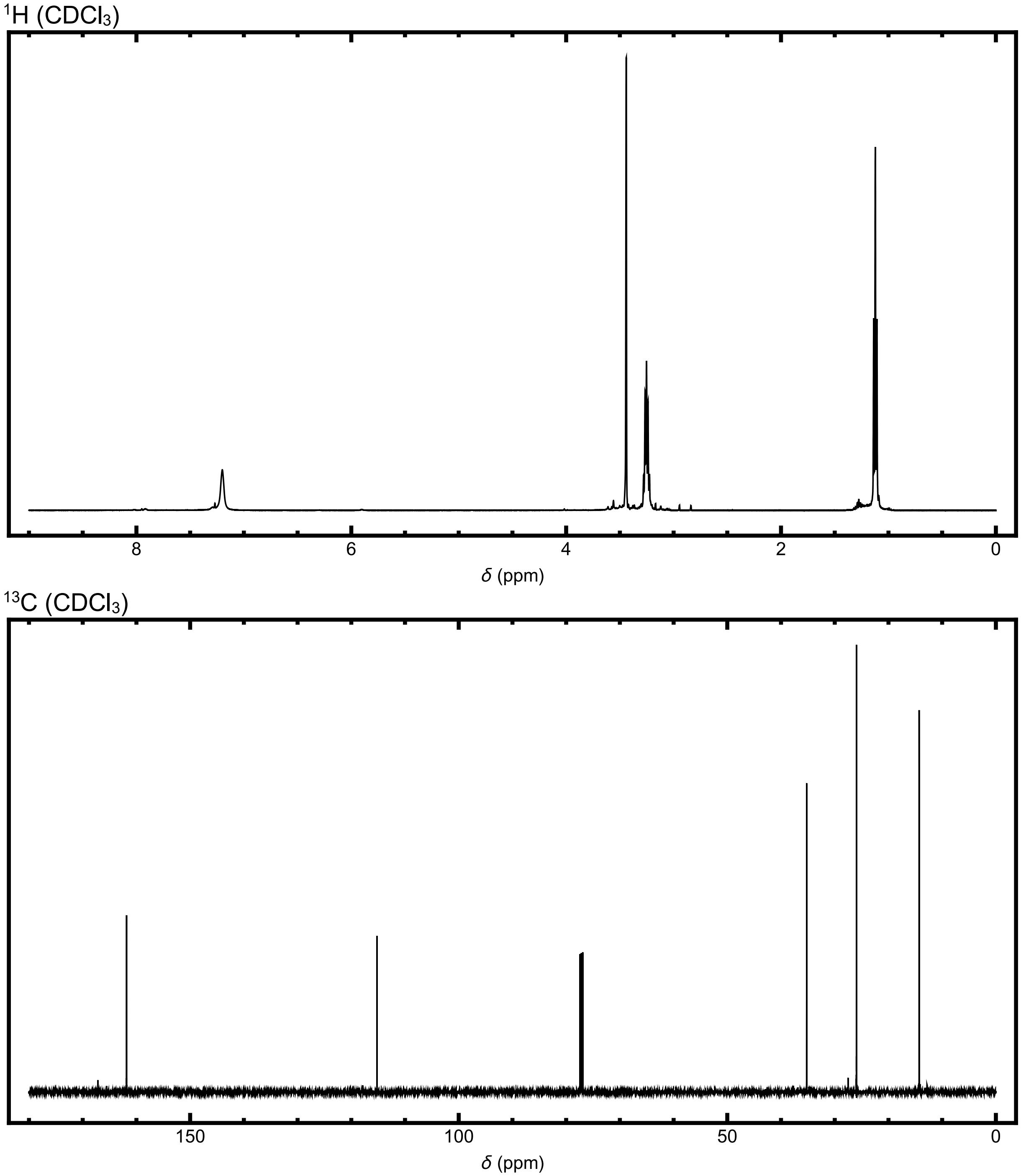

Figure S2. ^{1}H and ^{13}C NMR spectra for 2-cyano-N-ethylacetamide

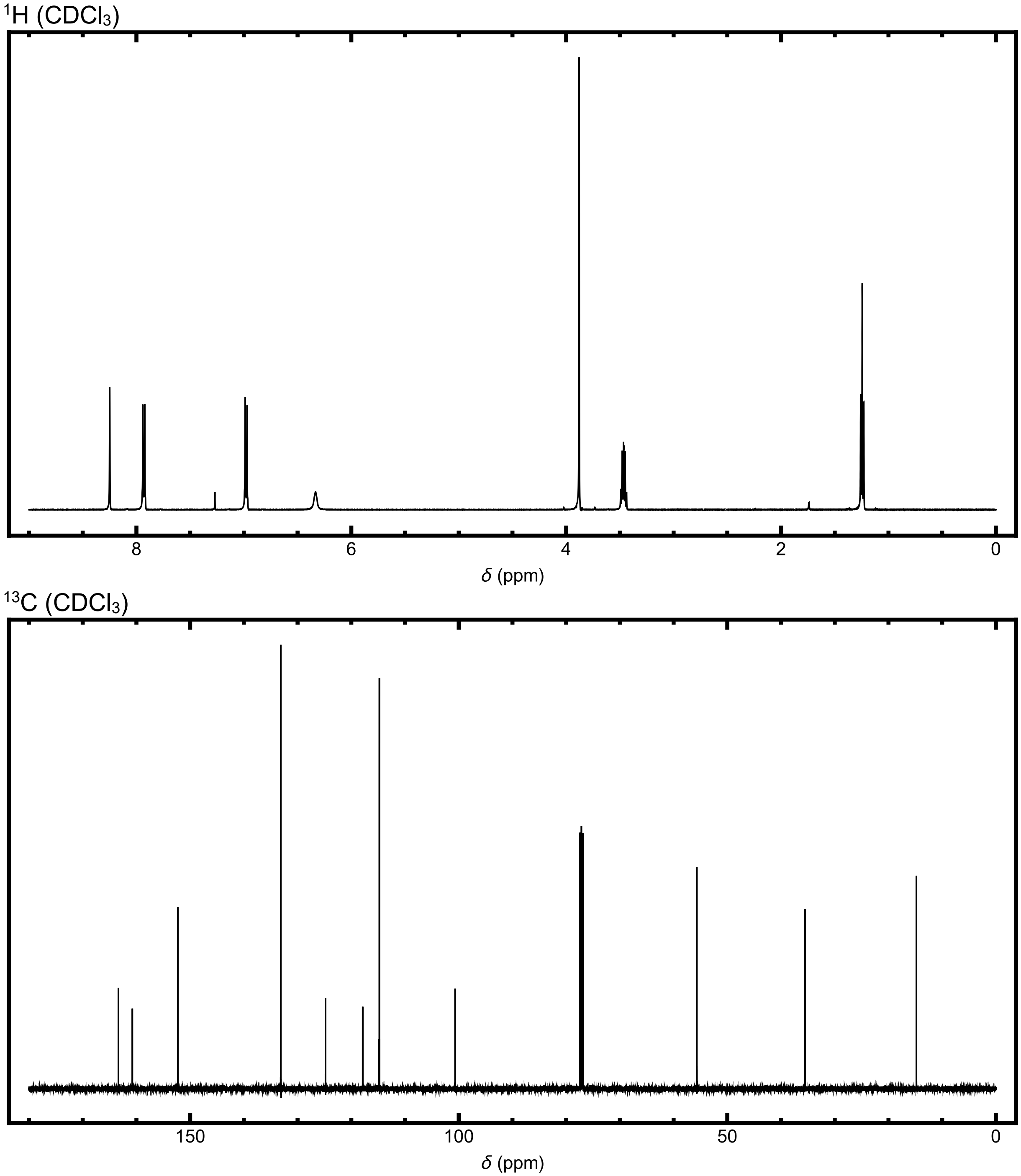

Figure S3. ^{1}H and ^{13}C NMR spectra for **2M**.

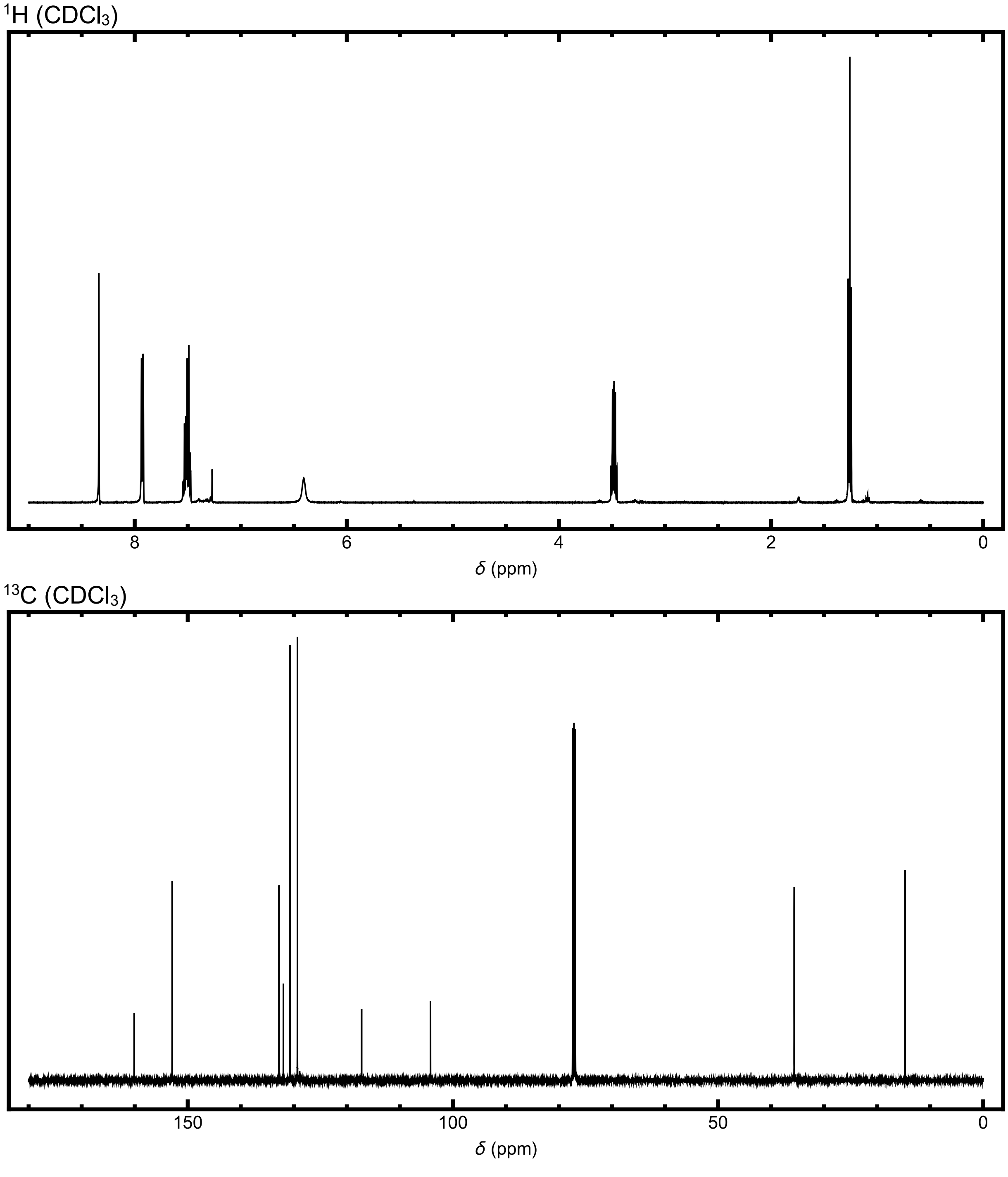

Figure S4. ^{1}H and ^{13}C NMR spectra for **2H**.

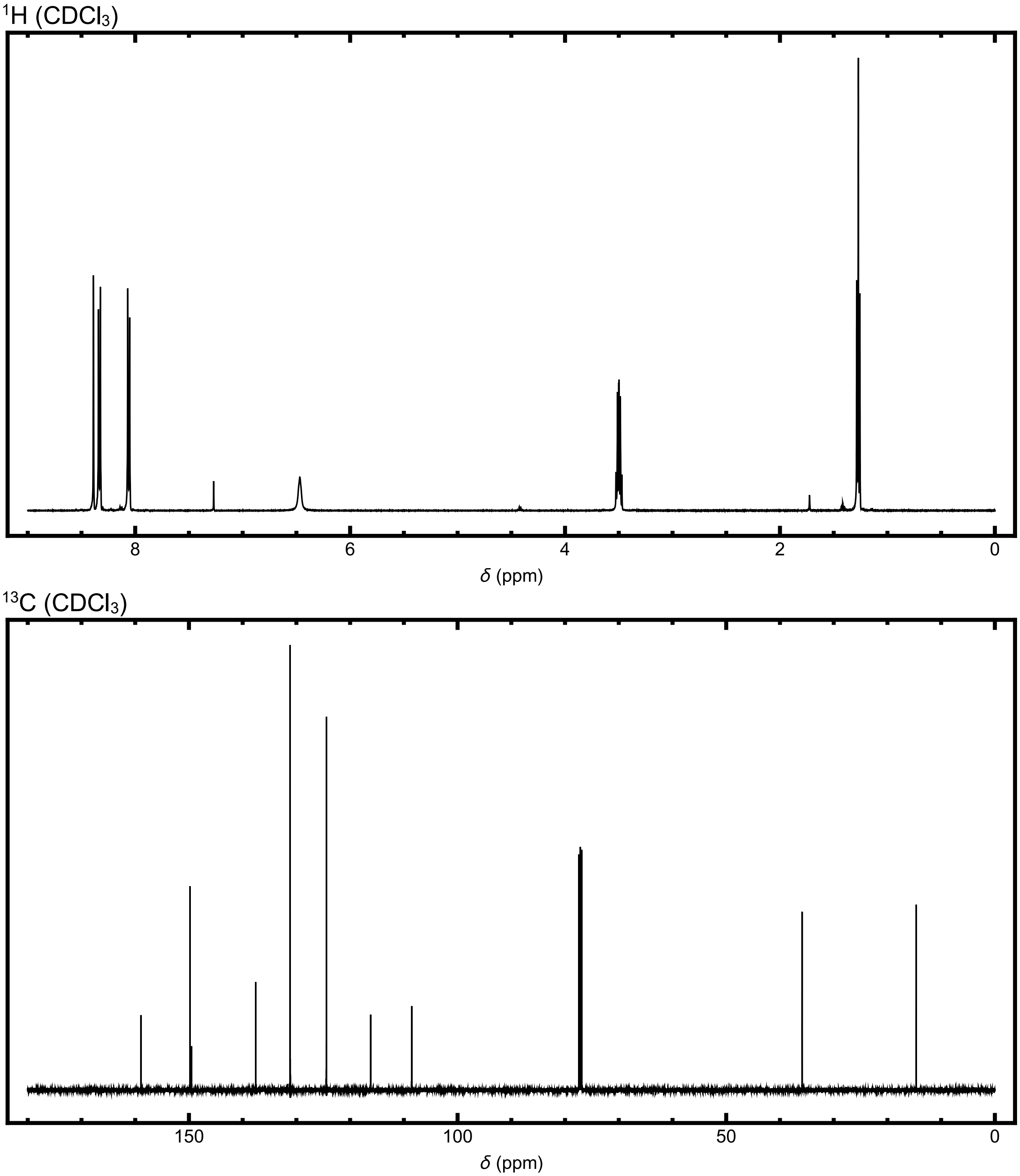

Figure S5. ^{1}H and ^{13}C NMR spectra for **2N**.

IB. Determination of K_{eq} from small molecule analogs (2M, 2H, 2N)

Stock solutions (200 mM, ~0.5 mL) of the benzalcyanoacetamide species of interest (**2M, 2H, 2N**) and 1-octanethiol were prepared in d_6-DMSO from freshly opened ampules in an inert (glovebox) environment. Equal volumes of each solution (255 μL) were then added to an intermediary vial and were vigorously mixed. Afterwards, the mixture was added to an NMR tube via a syringe and allowed to equilibrate for 12 hours. The sample was then introduced into the (preheated) NMR and allowed to equilibrate to the environmental temperature for 5 minutes before collecting data to ensure homogeneous sample temperature. Samples were monitored until equilibrium was reached (minimum equilibration time of 30 minutes). Afterwards, the temperature of the NMR would be increased, and the equilibration procedure would be repeated for all desired temperatures. Representative peaks of interest are displayed below in Figure S6, and the governing equation(s) used for monitoring adduct formation are also reported below.

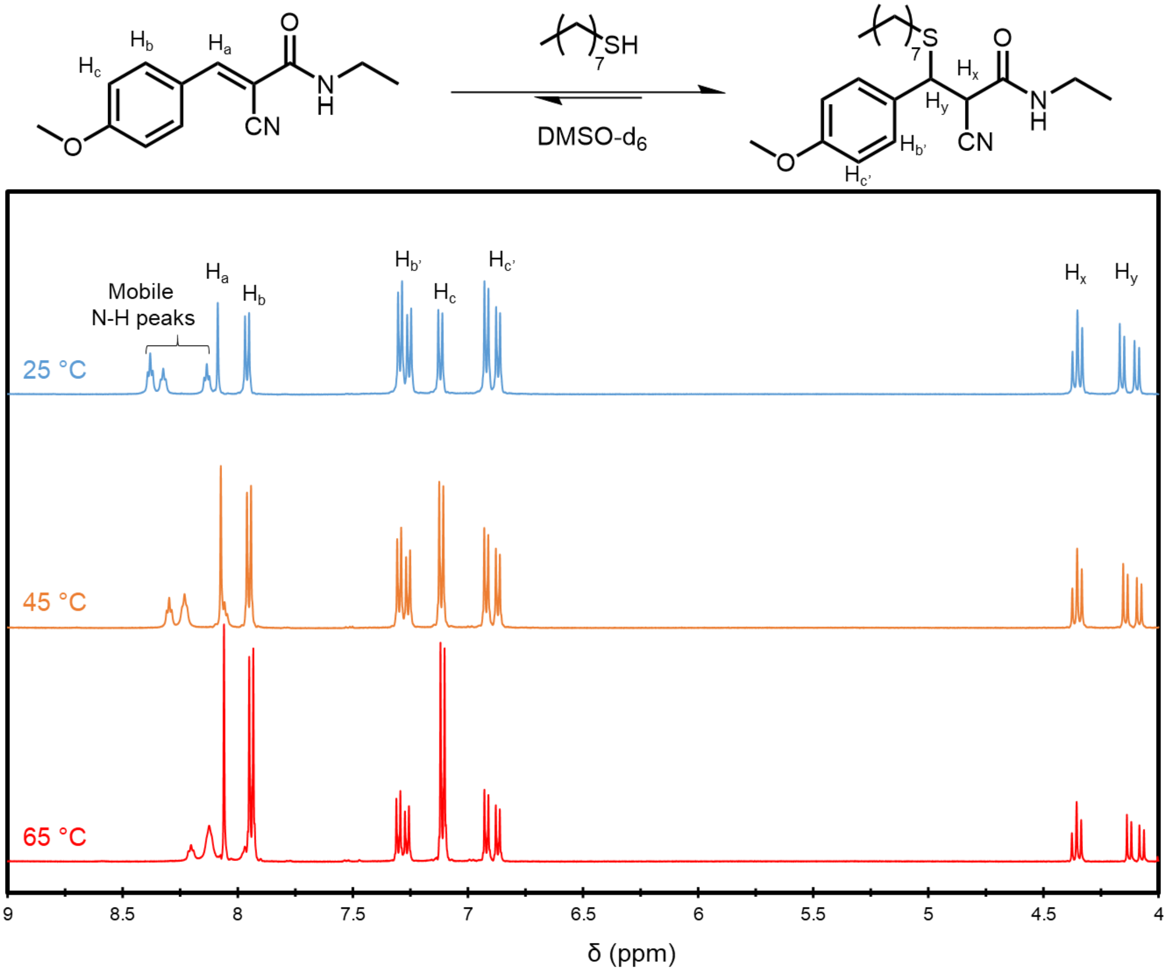

$$mol\,frac\,bound = \frac{\int H_x + \int H_y}{\int H_x + \int H_y + 2\int H_a} = \frac{\int H_x + \int H_y}{\int H_x + \int H_y + \int H_b} = \frac{\int H_x + \int H_y}{\int H_x + \int H_y + \int H_c}$$

$$K_{eq} = \frac{[bound\ species]}{[acceptor][thiol]} = \frac{sample\ concentration * (mol\,frac\,bound)}{(sample\ concentration * (1 - mol\,frac\,bound))^2}$$

Figure S6. Representative equilibrium NMR spectra for **2M** and 1-octanethiol obtained at several temperatures (100 mM in DMSO-d_6) along with formulas used to obtain the mole fraction (*mol frac*) of bonded species and K_{eq}. It is important to note that increasing temperature shifts the position of the N-H peaks, which will convolute integrated values, requiring the use of several peaks.

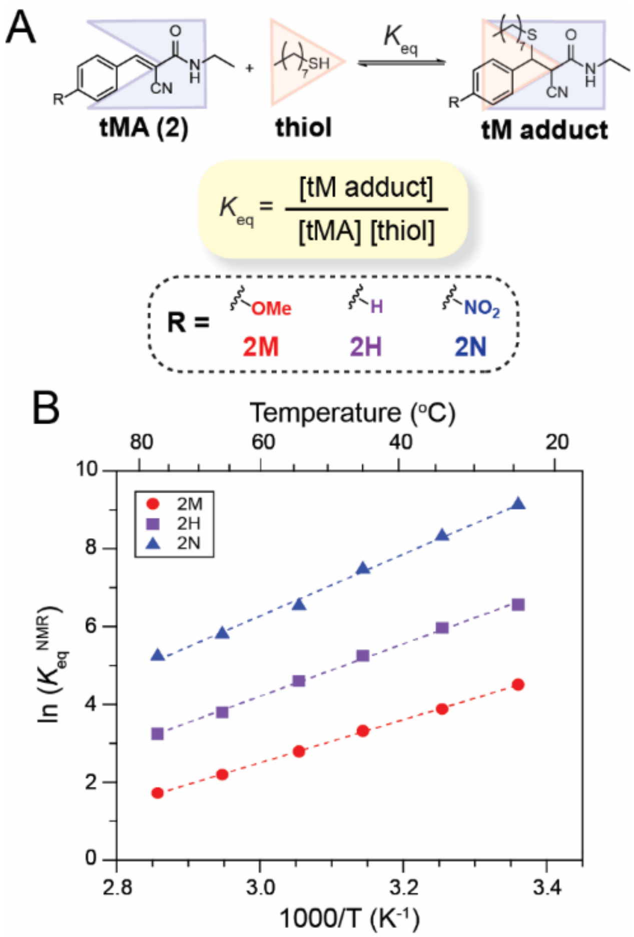

Figure S7. Small molecule analogs (**2M**, **2H**, **2N**) were used to determine the equilibrium bonding constant (K_{eq}^{NMR}) as a function of tMA chemistry and temperature. (*A*) Schematic of benzalcyanoacetamide electrophile (*tMA*) equilibrium reaction with 1-octanethiol (*thiol*) to form a thioether (*tM adduct*). (*B*) K_{eq}^{NMR} as a function of temperature and R-substituent at 100 mM equimolar conditions in DMSO. The dashed line is an Arrhenius fit to the data and the van't Hoff fit parameters are displayed in Table S2.

Table S2. Results of linear fits to the van't Hoff data (error reported from standard error of fit). See Herbert *et al.*[1] for additional details of K_{eq} measurement by NMR.

Compound	ΔH (kJ mol^{-1})	ΔS (J mol^{-1} K^{-1})
**2M**	-46.0 ± 0.4	-117 ± 1
**2H**	-56.0 ± 1.3	-133 ± 4
**2N**	-65.9 ± 2.1	-145 ± 7
**OH**	-33*	-132**

*Due to the presence of thiol groups as well as likely residual silanol groups on the particle surface, the ΔH value was estimated using the average hydrogen bond strength for O-H•••O and O-H•••S of alcohols and thiols as H-bond acceptors.[8]

This ΔS value was estimated as the average of the **2M, **2H**, and **2N** compounds.

II. Suspension Preparation

Thiol-functionalized particles and tMAs (**1M**, **1H**, **1N**, **3N**, or **3M**) or 4,000 g/mol hydroxy-terminated PPG (**OH**) were massed into 4 dram vials and homogenized by iterative hand mixing, centrifugation, and water bath sonication at 40 kHz and 130 W. Once homogeneous, the samples were annealed at 40 °C in the sonicator for 12 h and then allowed to rest for at least one week at ambient temperature prior to rheological characterization. Volume fractions were calculated using either $\rho_{2OH4000}$ = 1.004 g/mL or ρ_{tMA} = 1 g/mL and the experimentally determined value of $\rho_{Particles}$ = 1.92 g/mL.

Estimation of Fraction Bonded SH (*p*) for DCSs from K_{eq}^{NMR}:

The equation for the equilibrium association constant (K_{eq}^{NMR}) is given by Equation 1:

$$K_{eq}^{NMR} = \frac{[tM\ adduct]}{[free\ SH][free\ tMA]} \tag{1}$$

As K_{eq}^{NMR} was determined under equimolar conditions, it cannot directly be related to the mole fraction of bound thiol (*p*) in **DCSs** (which are at stoichiometric imbalance where the thiol (SH) is the limiting reagent) as indicated by the equation in Figure S6. Instead, the concentrations of the various components are given by Equations 2-4:

$$[tM\ adduct] = [SH]_0 * p \tag{2}$$

$$[free\ SH] = [SH]_0 * (1 - p) \tag{3}$$

$$[free\ tMA] = [tMA]_0 - [tM\ adduct] = [tMA]_0 - [SH]_0 * p \tag{4}$$

Where $[SH]_0$ and $[tMA]_0$ correspond to the initial concentrations of 0.016 and 0.46 M in **DCSs**, respectively. Substituting Equations 2-4 into Equation 1 yields Equation 5:

$$K_{eq}^{NMR} = \frac{p}{(1-p)*([tMA]_0-[SH]_0*p)} \tag{5}$$

Using the K_{eq}^{NMR} values, this equation was solved analytically to yield *p* and is plotted as the solid curve in Figure 3C in the main text.

III. Rheological Characterization

All rheological measurements were conducted using either an Anton Paar MCR301 or MCR702 rheometer with a smooth parallel plate geometry (d = 25 mm) using a gap size of approximately 0.1 mm. Temperature was controlled with a precision of 0.1 °C using a Peltier device and water circulator.

The apparent viscosity of the suspensions measured by the rheometer ($\eta_{apparent}$) at each shear rate or shear stress was divided by that of the Newtonian background solvent (η_0, Figure S8) to attain the reduced viscosity (η_r) and thereby isolate the viscosity contribution of the particles.

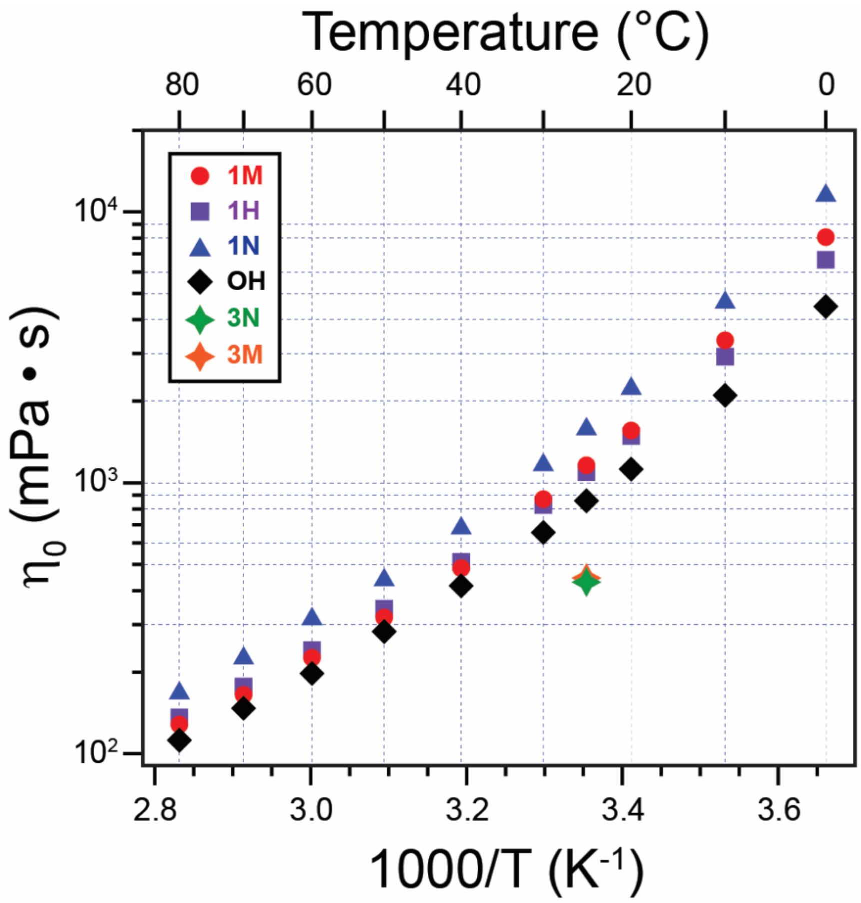

Figure S8. Viscosity (η_0) of ditopic tMAs (**1M**, **1H**, **1N**), monotopic tMA (**3M, 3N**), and hydroxy-terminated 4000 g/mol polypropylene glycol (**OH**) as a function of temperature. The viscosities were Newtonian over the measured shear rate range of 0.1 – 100 s^{-1}. Error bars were calculated from the standard deviation in the forward and backward ramps and are smaller than the marker size.

For the hysteresis loop experiments, a preshear of either 20 s^{-1} (data in Figure 2) or 5 s^{-1} (data in Figures 3 & 4) were applied for 120 s, followed by a rest period of 300 s and then the shear rate sweep was conducted. The data shown are the average of 3 consecutive forward or backward shear rate ramps with an intervening preshear and rest period. The error bars represent the standard deviation of the viscosity.

For the temperature-dependent measurements, the samples were equilibrated at each temperature for 600 s with ω = 10 Hz and γ = 0.5%. The oscillatory measurements were conducted from ω = 100 to 0.1 Hz with γ = 0.5%, which took approximately 1800 s, and then the hysteresis loop measurements were performed in triplicate with a waiting time of 10 s/pt. Temperature dependent rheology exhibited qualitatively the same rheological behavior on cooling.

For the constant shear rate or constant shear stress (*creep*) measurements shown in Figure 2 and Figures S11-13, a preshear of 20 s^{-1} was applied for 120 s and then the shear rate or stress was immediately reduced to the indicated value. The data were collected at 700 logarithmically spaced points, starting with 0.1 s/pt and ending with a collection time of 100 s for each data point. From these data, the viscosity was averaged using a floating window of 40 bins (~4 s at the shortest times and ~4000 s at the longest times) and the error bars represent the standard deviation of the viscosity.

Rheological Measurement Limits
There are a number of measurement artefacts in rheometry which could potentially contribute to spurious data and define a “measurement window” in which data is believable.[9] These various limits in relation to our experimentally measured data are depicted in Figure S9, showing that these data lie safely within as region bounded by the minimum shear rate, low/high torque limit, and inertial sample ejection. The secondary flow limit and high torque limit are off the scale of the graph.

Measurements at different gap heights revealed the same trends in terms of a temperature-dependent transition from antithixotropy to shear thinning (or lack thereof). Thus while wall slip could potentially lead to lower measured viscosities, particularly at low shear rates for shear thinning samples, it does not change any of the qualitative trends or main conclusions within the manuscript.

Finally, the highest particle Reynold’s number reached in our experiments, which occurred at the highest temperatures where the dynamic oil viscosity was the lowest, was $10^{-6} <<<<< 1$, meaning that particle inertia can be safely neglected.

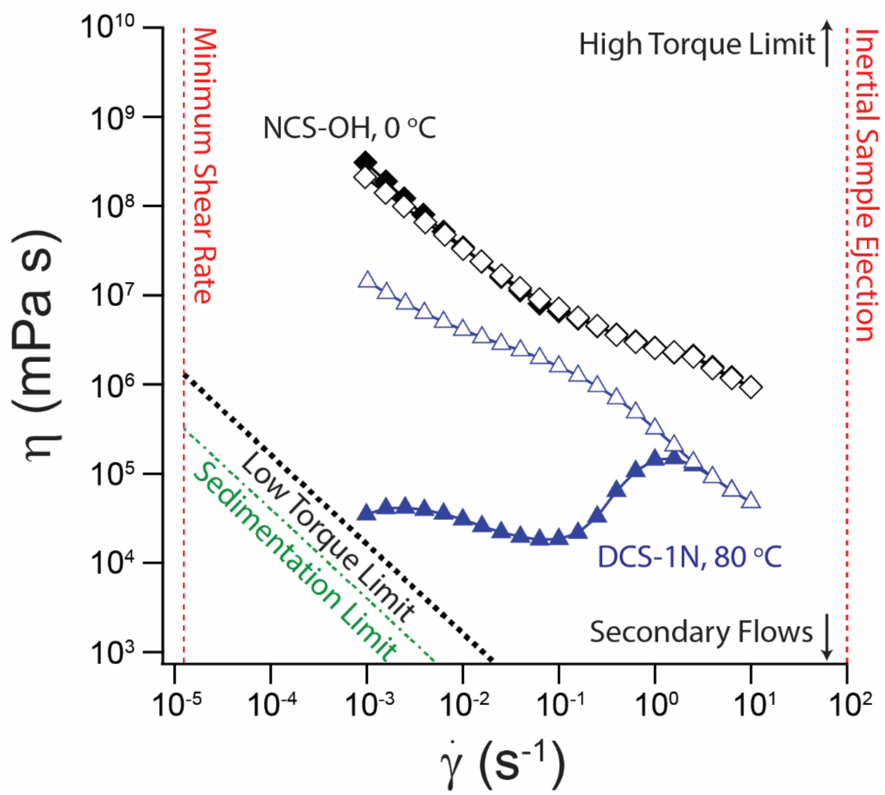

Figure S9. Identifying the measurable limits of shear rheometry for a 25 mm parallel plate geometry with a gap of 0.1 mm in comparison to the highest (**NCS-OH** at 0 °C) and lowest (**DCS-1N** at 80 °C) measured viscosities in this study. The low/high torque limits and secondary flow limit (*below the scale of the graph,* $Re_{max} \sim 10^{-6}$) were calculated following Ewoldt *et al.*[9] The sedimentation lower stress limit[10] is below the low torque limit for these measuring conditions. Inertial sample ejection was observed for $\dot{\gamma} > 100$ s^{-1} and the minimum shear rate was determined from the instrumental specifications.

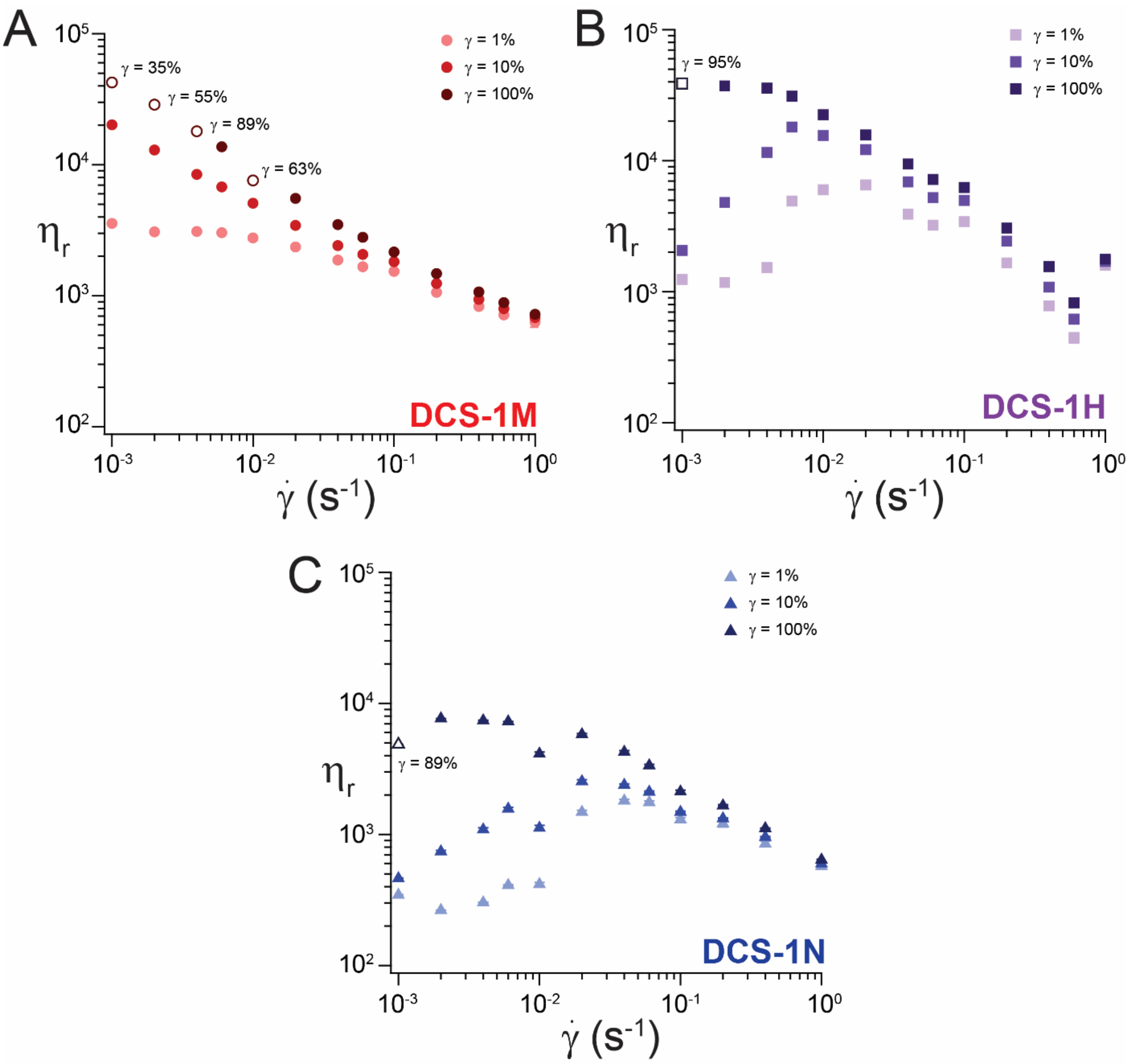

Figure S10. Demonstrating how viscosity evolves with strain while holding at a constant shear rate. Data were taken from constant shear rate experiments shown in Figures 2B, S12, and S13 and the error bars (oftentimes smaller than the marker size) represent the standard deviation of the viscosity as described above.

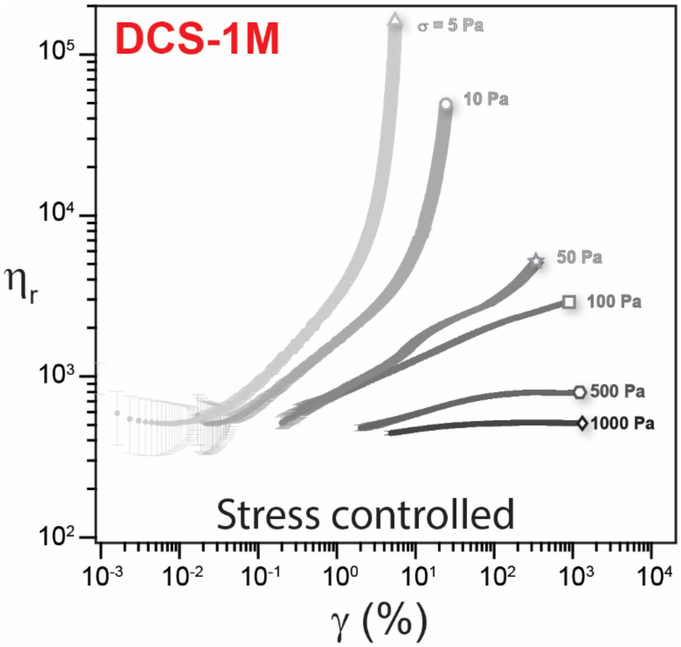

Figure S11. Constant stress (*creep*) measurements of **DCS-1M** at 25 °C showing the change in the reduced viscosity (η_r) as a function of strain (γ). The applied preshear was 20 s^{-1} for 120 s and then the shear stress (σ) was immediately set to the indicated value. The system exhibits a viscosity bifurcation,[11] wherein the viscosity diverges for $\sigma \leq 10$ Pa and flows for $\sigma \geq 100$ Pa. Error bars represent the standard deviation and were calculated using a floating window as described above. All data are above the minimum shear rate limit.

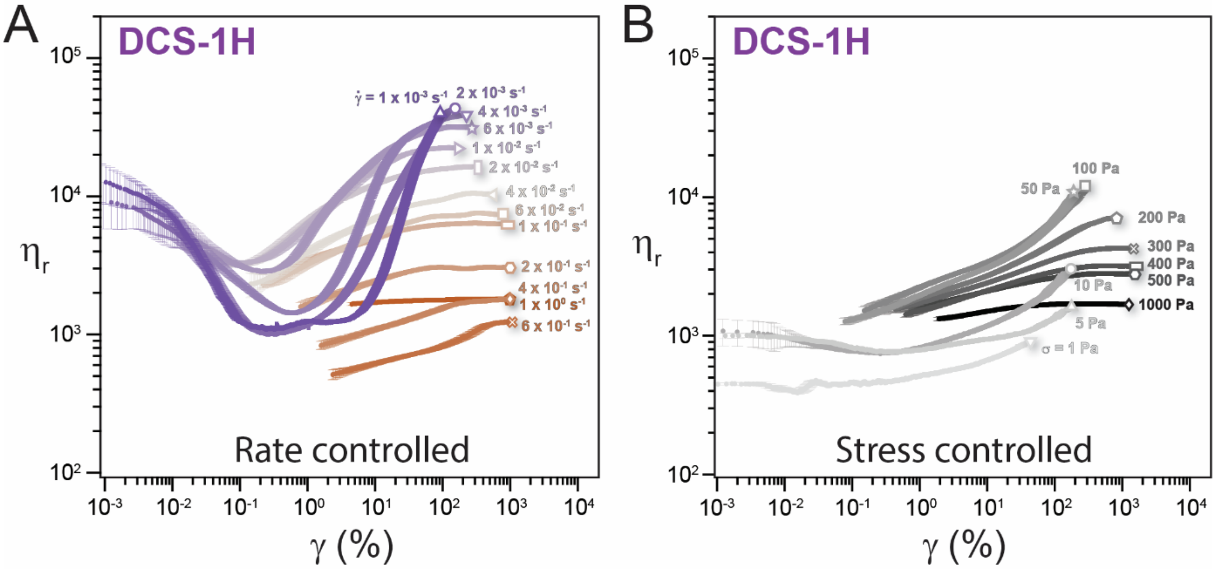

Figure S12. (*A*) Constant shear rate and measurements of **DCS-1H** at 25 °C. Reduced viscosity (η_r) increases with strain (γ) and at most rates plateaus to a high γ viscosity value. These high γ values are plotted in Figure 3A as the 25 °C high γ values for **DCS-1H**. (*B*) Constant shear stress (*creep*) measurements show a viscosity bifurcation. Error bars for both plots were calculated as described above and all stress-controlled data are above the minimum shear rate limit.

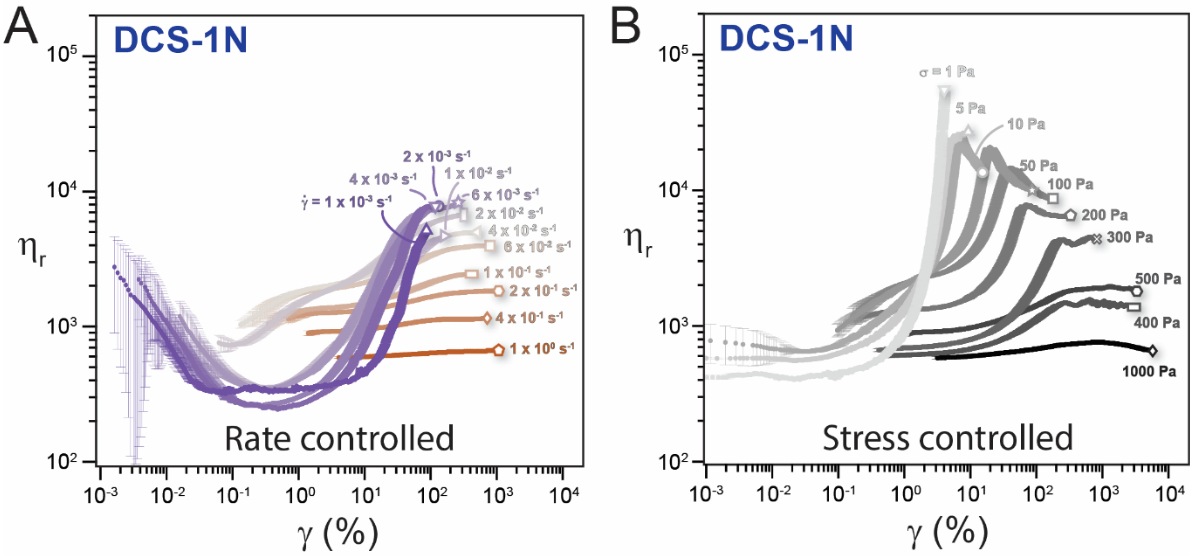

Figure S13. (*A*) Constant shear rate and measurements of **DCS-1N** at 25 °C. Reduced viscosity (η_r) increases with strain (γ) and plateaus to a high γ viscosity value. These high γ values are plotted in Figure 3A as the 25 °C high γ values for **DCS-1N**. (*B*) Constant shear stress (*creep*) measurements also showing a viscosity bifurcation for $\sigma \leq 1$ Pa. It is currently unknown why this system exhibits stress overshoots for $\sigma \geq 10$ Pa. Error bars were calculated as described above and all stress-controlled data are above the minimum shear rate limit.

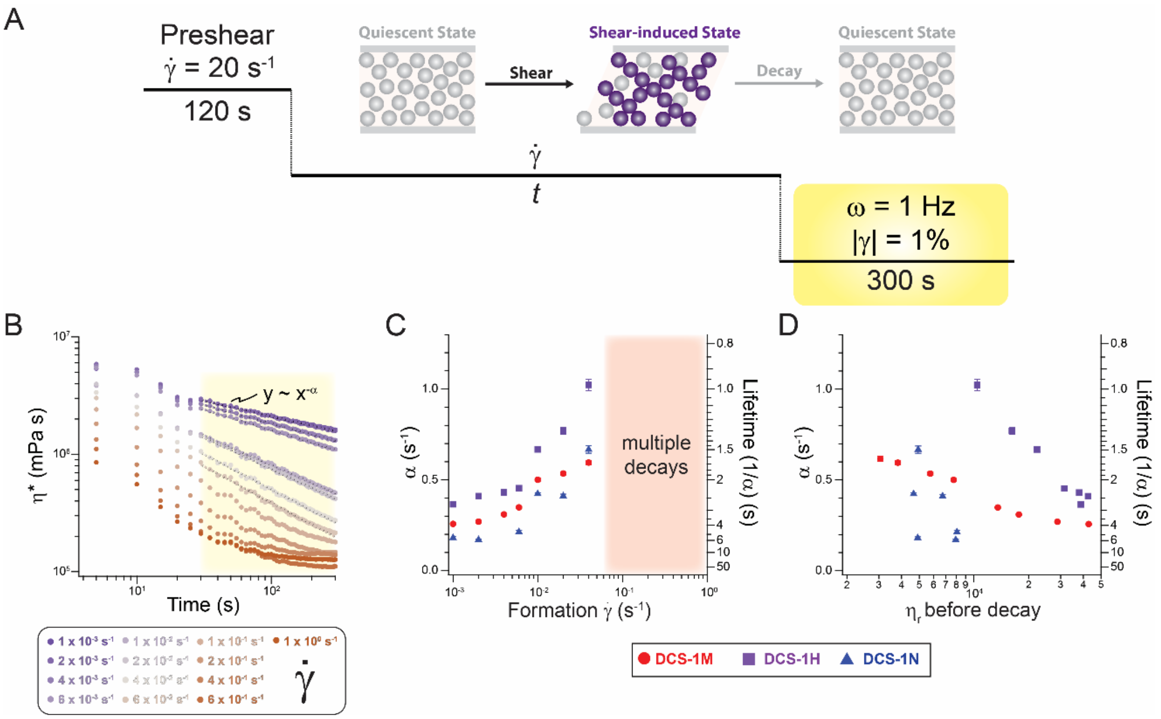

Figure S14. Oscillatory decay of the high viscosity antithixotropic state. (*A*) Experimental protocol: after an initial preshear, a constant shear rate was applied as shown in Figure 2 for **DCS-1M** and Figures S12-13 for **DCS-1H** and **DCS-1N**, followed by 300 s of oscillation at ω = 1 Hz and |γ| = 1%. (*B*) Representative decay of η* for **DCS-1M** networks constructed at different shear rates. (*C*) Power law exponent extracted from *t* = 30 – 300 s showing that structures formed at lower shear rates decay more slowly. (*D*) Power law exponent plotted against the particle network viscosity prior to shear cessation (η_r). Error bars are the standard error associated with the power law fitting.

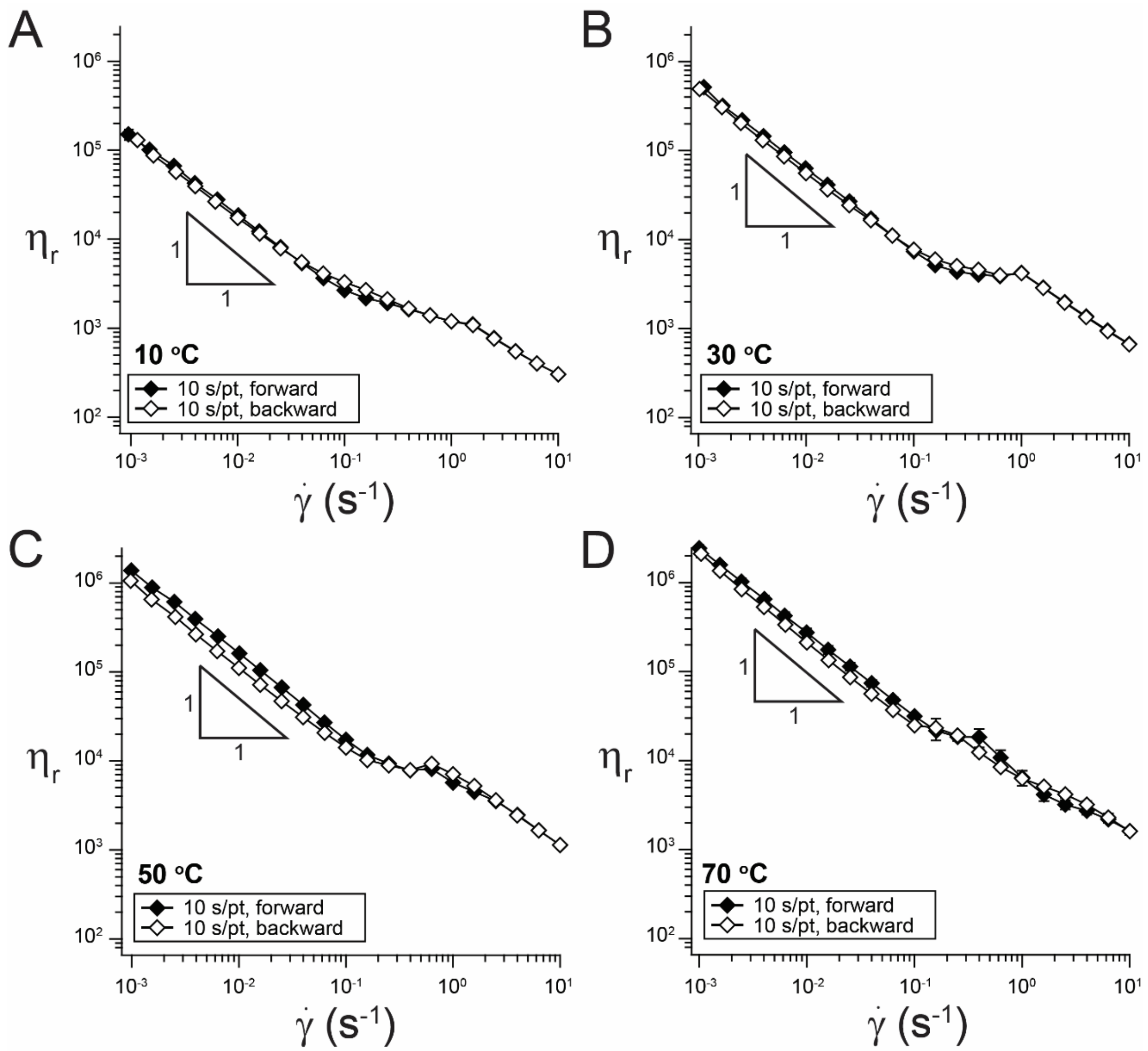

Figure S15. Temperature dependent shear rate ramps for **NCS-OH** showing shear thinning that is either reversible or with a small amount of thixotropy over the entire temperature range. The error bars are on the order of the marker size and represent the standard deviation of 3 forward or backward shear rate sweeps.

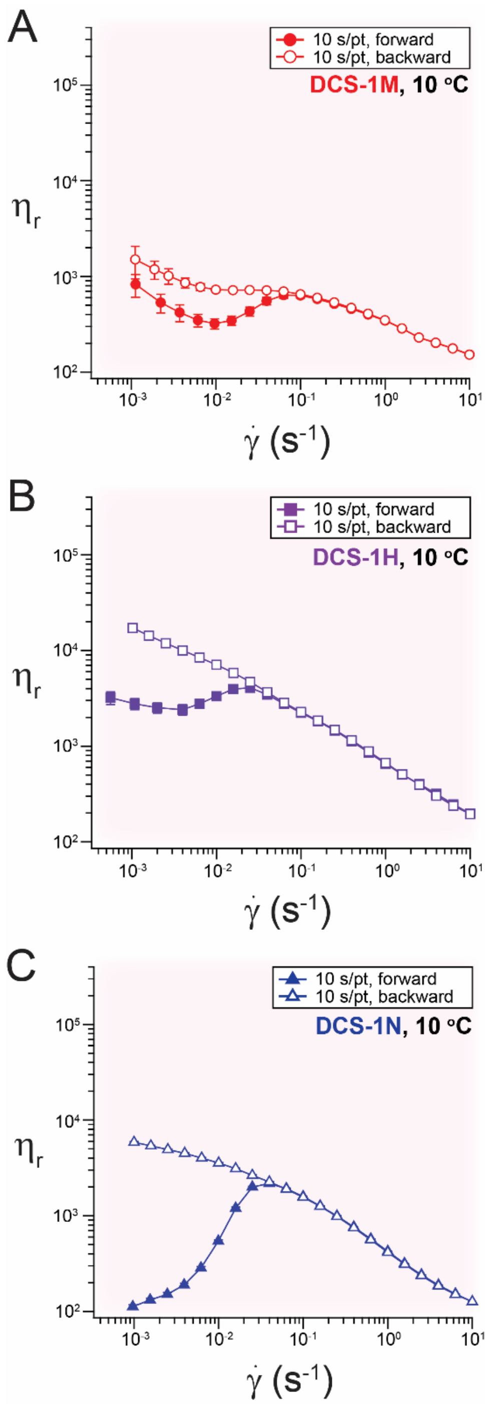

Figure S16. Hysteresis loops shows antithixotropy for all **DCSs** at 10 °C. Error bars represent the standard deviation of 3 forward or backward shear rate sweeps.

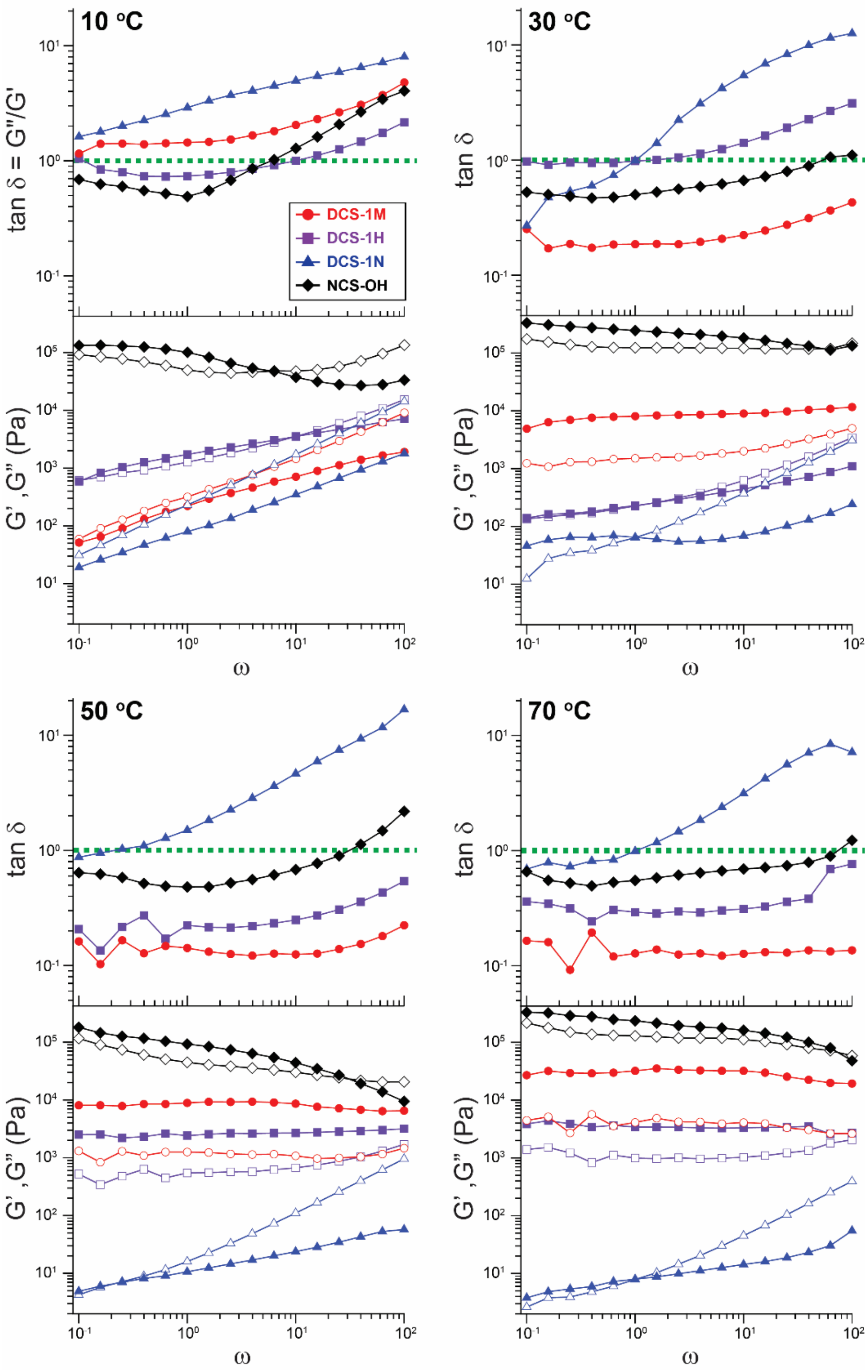

Figure S17. Temperature dependent small amplitude oscillatory shear (SAOS) measurements of **DCSs** and **NCS-OH**. Measurements conducted within the linear viscoelastic regime with $|\gamma|$ = 0.5%. Heating **DCS-1M** shows a transition from liquid-like ($\tan\delta > 1$) at 10 °C to solid-like ($\tan\delta < 1$) ≥ 30 °C. This transition occurs between 40 °C and 50 °C for **DCS-1H**, whereas **DCS-1N** is liquid-like or viscoelastic ($\tan\delta$ crosses 1 within the frequency range) at all temperatures.

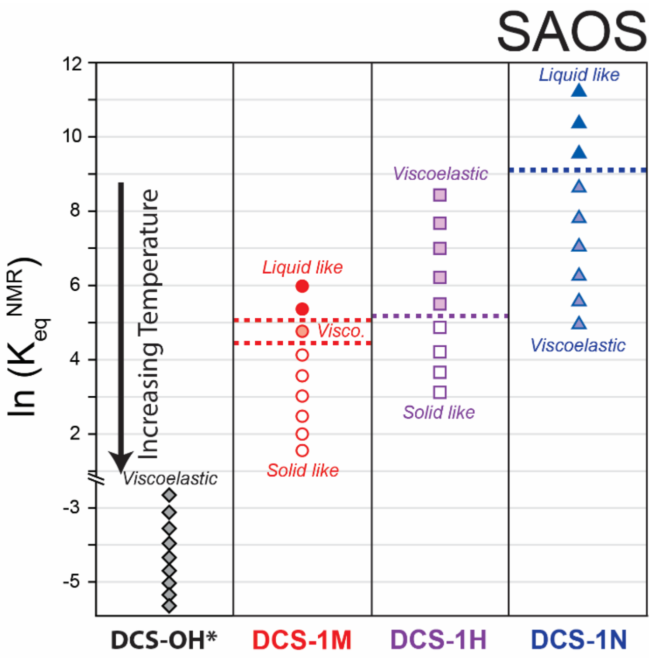

Figure S18. SAOS rheological state diagram as a function of K_{eq}^{NMR} for 0 – 80 °C for each system from the data shown in Figure S17. This indicates the quiescent state of the suspensions, whereas the rheological state diagram shown in Figure 3B of the main text is for non-linear steady shear rheology.

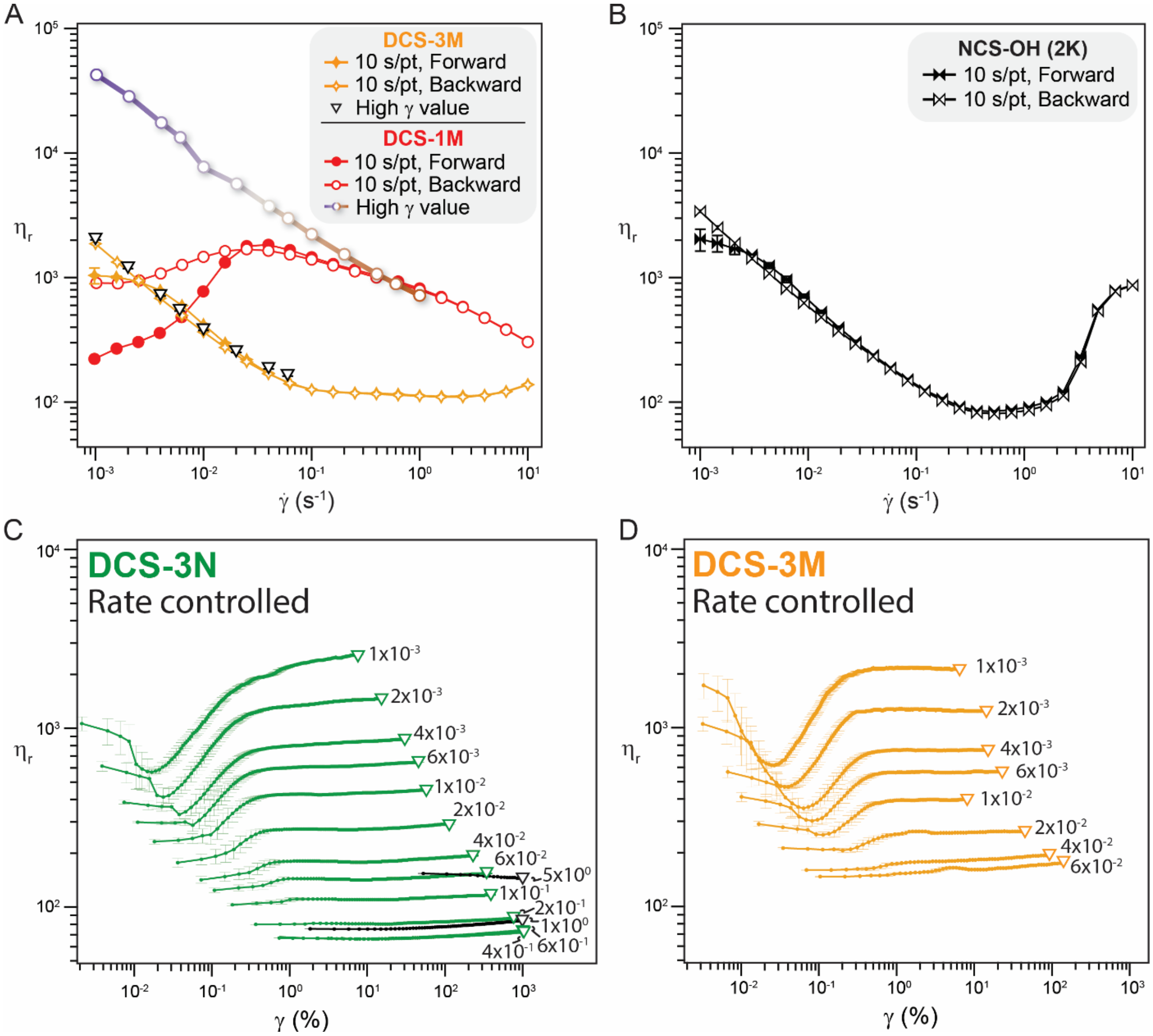

Figure S19. (*A*) Comparing **DCSs** with monotopic tMA **3M** and ditopic **1M**. Reduced viscosity (η_r) for a forward-backward shear rate ($\dot{\gamma}$) ramp reveals mostly reversible behavior for **DCS-3M**, with a backward shear rate ramp which nearly matches the high γ viscosity values. This behavior contrasts greatly with that of the **DCS-1M**, which shows pronounced hysteresis. The high γ reduced viscosity for **DCS-3M** is also much lower than that of **DCS-1M** at a given shear rate. The error bars are on the order of the marker size and represent the standard deviation of 3 forward or backward shear rate sweeps. (*B*) Demonstrating that the slight hysteresis at low shear rates (~10^{-3} s^{-1}) seen for **DCS-3M** and **DCS-3N** is also observed for a non-covalent suspension prepared with dihydroxy poly(propylene glycol) with M_n ~2,000 g/mol **NCS-OH (2K)**. This feature is likely related to an inhomogeneous flow profile at short times.[12] (*C*) Constant shear rate measurements for **DCS-3N** showing that η_r increases with strain (γ) and plateaus to a high γ viscosity value. **DCS-3N** requires far less strain to equilibrate compared to **DCS-1N** (Figure S13). The high γ values for **DCS-3N** are plotted in Figure 4B as the high γ values. (*D*) Constant shear rate measurements for **DCS-3M**, which also equilibrate at much lower strain values than **DCS-1M**. All data shown are for 25 °C.

IV. References

1. Herbert, K. M.; Getty, P. T.; Dolinski, N. D.; Hertzog, J. E.; de Jong, D.; Lettow, J. H.; Romulus, J.; Onorato, J. W.; Foster, E. M.; Rowan, S. J. Dynamic Reaction-Induced Phase Separation in Tunable, Adaptive Covalent Networks. *Chem. Sci.* **2020,** *11*, 5028-5036.
2. Ammann, C.; Meier, P.; Merbach, A. A Simple Multinuclear NMR Thermometer. *J. Magn. Reson.* **1982,** *46*, 319-321.
3. Crucho, C. I. C.; Baleizão, C.; Farinha, J. P. S. Functional Group Coverage and Conversion Quantification in Nanostructured Silica by ^{1}H NMR. *Anal. Chem.* **2017,** *89*, 681-687.
4. Estephan, Z. G.; Jaber, J. A.; Schlenoff, J. B. Zwitterion-Stabilized Silica Nanoparticles: Toward Nonstick Nano. *Langmuir* **2010,** *26*, 16884-16889.
5. Zhuravlev, L. T. Concentration of Hydroxyl Groups on the Surface of Amorphous Silicas. *Langmuir* **1987,** *3*, 316-318.
6. Cope, A. C. Condensation Reactions. I. The Condensation of Ketones with Cyanoacetic Esters and the Mechanism of the Knoevenagel Reaction. *J. Am. Chem. Soc.* **1937,** *59*, 2327-2330.
7. Patent EP147718A1 November 17, 2004.
8. Biswal, H. S.; Shirhatti, P. R.; Wategaonkar, S. O−H···O Versus O−H···S Hydrogen Bonding I: Experimental and Computational Studies on the *p*-Cresol·H_2O and *p*-Cresol·H_2S Complexes. *J. Phys. Chem. A* **2009,** *113*, 5633-5643.
9. Ewoldt, R. H.; Johnston, M. T.; Caretta, L. M., Experimental Challenges of Shear Rheology: How to Avoid Bad Data. In *Complex Fluids in Biological Systems: Experiment, Theory, and Computation*, Spagnolie, S. E., Ed. Springer New York: New York, NY, 2015; pp 207-241.
10. Richards, J. A.; O'Neill, R. E.; Poon, W. C. K. Turning a Yield-Stress Calcite Suspension into a Shear-Thickening One by Tuning Inter-Particle Friction. *Rheol. Acta* **2020**.
11. Ovarlez, G.; Tocquer, L.; Bertrand, F.; Coussot, P. Rheopexy and Tunable Yield Stress of Carbon Black Suspensions. *Soft Matter* **2013,** *9*, 5540-5549.
12. Wagner, C. E.; Barbati, A. C.; Engmann, J.; Burbidge, A. S.; McKinley, G. H. Apparent Shear Thickening at Low Shear Rates in Polymer Solutions Can Be an Artifact of Non-Equilibration. *Appl. Rheol.* **2016,** *26*, 36-40.
